# Heme Oxygenase-1 Predicts Risk Stratification and Immunotherapy Efficacy in Lower Grade Gliomas

**DOI:** 10.3389/fcell.2021.760800

**Published:** 2021-11-09

**Authors:** Wenrui Ye, Zhixiong Liu, Fangkun Liu, Cong Luo

**Affiliations:** ^1^Department of Neurosurgery, Xiangya Hospital, Central South University (CSU), Changsha, China; ^2^National Clinical Research Center for Geriatric Disorders, Xiangya Hospital, Central South University (CSU), Changsha, China; ^3^Department of Urology, Xiangya Hospital, Central South University (CSU), Changsha, China

**Keywords:** glioma, heme oxygenase-1, prognosis, risk signature, single-cell sequencing

## Abstract

**Background:** Gliomas are the most common tumors in human brains with unpleasing outcomes. Heme oxygenase-1 (*HMOX1*, *HO-1*) was a potential target for human cancers. However, their relationship remains incompletely discussed.

**Methods:** We employed a total of 952 lower grade glioma (LGG) patients from TCGA and CGGA databases, and 29 samples in our hospital for subsequent analyses. Expression, mutational, survival, and immune profiles of *HMOX1* were comprehensively evaluated. We constructed a risk signature using the LASSO Cox regression model, and further generated a nomogram model to predict survival of LGG patients. Single-cell transcriptomic sequencing data were also employed to investigated the role of *HMOX1* in cancer cells.

**Results:** We found that *HMOX1* was overexpressed and was related to poorer survival in gliomas. *HMOX1*-related genes (HRGs) were involved in immune-related pathways. Patients in the high-risk group exhibited significantly poorer overall survival. The risk score was positively correlated with the abundance of resting memory CD4+ T cells, M1, M2 macrophages, and activated dendritic cells. Additionally, immunotherapy showed potent efficacy in low-risk group. And patients with lower *HMOX1* expression were predicted to have better response to immunotherapies, suggesting that immunotherapies combined with *HMOX1* inhibition may execute good responses. Moreover, significant correlations were found between *HMOX1* expression and single-cell functional states including angiogenesis, hypoxia, and metastasis. Finally, we constructed a nomogram which could predict 1-, 3-, and 5-year survival in LGG patients.

**Conclusion:** *HMOX1* is involved in immune infiltration and predicts poor survival in patients with lower grade glioma. Importantly, *HMOX1* were related to oncological functional states including angiogenesis, hypoxia, and metastasis. A nomogram integrated with the risk signature was obtained to robustly predict glioma patient outcomes, with the potential to guide clinical decision-making.

## Introduction

Gliomas, originating from intrinsic constituent cells of the brain, are the most common primary tumors there (Sanai et al., 2005). Before genome-wide molecular profiling researches revealed the comprehensive genomic landscape of human gliomas (Brat et al., 2015; Suzuki et al., 2015), glioma classification was largely based on their microscopic and immunohistochemical features. Development at the molecular biology level identifies novel biomarkers for optimized classification strategy and promising treatment targets. Predictive biomarkers recognized and used in clinics mainly included isocitrate dehydrogenase (IDH) mutation, the discovery of which constituted a key breakthrough in the understanding of WHO grade II/III gliomas (Yan et al., 2009). Besides, the presence of O6-methylguanine-DNA methyltransferase (MGMT) promoter methylation predicts benefit from temozolomide-based chemotherapy in patients with IDH-wildtype glioma (Wick et al., 2012). Furthermore, 1p/19q codeletion is predictive for benefit from combined radiotherapy and chemotherapy (with procarbazine, lomustine, and vincristine) in two phase III trials (Cairncross et al., 2013; van den Bent et al., 2013). Novel pathogenesis-based treatments targeting oncogenic signaling pathways such as BRAF mutation (Robinson et al., 2014), epidermal growth factor receptor (EGFR) amplification (Phillips et al., 2016), and fibroblast growth factor receptor (FGFR)-TACC fusion (Stefano et al., 2015) harbored the potential for LGG treatment.

Although these molecular subclassifications deepened our understanding of tumorigenesis and personalized therapeutics, a certain LGG population still acquired resistance to these targeted therapies. Furthermore, gliomas are not considered highly immunogenic due to the low mutational loads, besides they are featured by severe immunosuppression mediated by immune-inhibitory factors, such as programmed cell-death 1 ligand 1 (PDCD1LG1) and secreted transforming growth factor β (Nduom et al., 2015). Therefore, we hope that newly identified biomarkers can overcome immunosuppression, exploit antitumor immune responses, and guide individualized treatments.

Heme oxygenase is an essential enzyme in heme catabolism as it cleaves cellular heme to form biliverdin. *HMOX1* overexpression is observed in various solid malignancies, including bladder (Miyata et al., 2014), breast (Noh et al., 2013), colon (Yin et al., 2014), glioma (Gandini et al., 2014), lung (Degese et al., 2012), prostate (Maines and Abrahamsson, 1996), and gastric (Yin et al., 2012), cancers. Although HMOX1 prevents DNA damage under normal conditions, *HMOX1* overexpression paradoxically promotes cancer cell proliferation and invasiveness at late phase of tumorigenesis (Was et al., 2006; Ph et al., 2013). Targeting HMOX1 was effective for hormone-refractory prostate cancer (Alaoui-Jamali et al., 2009) and it has been shown to reverse imatinib resistance in myeloid leukemia (Mayerhofer et al., 2008). Several imidazole-based non-porphyrin HMOX1 inhibitors were recently developed, which exhibited selectivity toward HMOX1 (Pittalà et al., 2013). Meanwhile, they showed potent anti-tumor activities both *in vitro* and *in vivo*, with the potential for clinical applications (Salerno et al., 2013). In a nutshell, these findings shed a light on the future for targeting HMOX1 to promote cancer immunotherapy.

Currently, several clinical trials are attempting to explore the clinical benefits of targeting HMOX1 (or related molecules) in the treatment of lower grade gliomas and other solid cancers (Supplementary Table 1). We comprehensively analyzed two independent glioma cohorts, as well as samples from our institution, to explore the *HMOX1* profiling in the context of gliomas. In addition, an HRG-based risk signature was established to predict the outcome of patients diagnosed with primary lower grade gliomas. The multifaceted performance of the IIRS was also examined to reveal its superior predictive ability of response to immunotherapy.

## Materials and Methods

### Data Extraction

Transcriptomic, copy number variations (CNV), and clinical data were extracted from The Cancer Genome Atlas (TCGA) and Chinese Glioma Genome Atlas (CGGA) databases, and 952 samples with lower grade glioma were finally included. 508 samples extracted from the TCGA database were defined as the training set, while 444 from the CGGA database were set as the validation set. Normal or glioblastoma (GBM, WHO grade IV glioma) samples were excluded.

### Expression, Mutational, and Survival Analysis

We evaluated the expression distribution of *HMOX1* between tumor and normal tissues in TCGA pan-cancers. *HMOX1* expression between different clinic-related subgroups were explored in both cohorts. The associations between *HMOX1* expression and patient outcomes, including overall survival (OS), disease specific survival (DSS), and progression free interval (PFI), in TCGA pan-cancer sets were evaluated using univariate Cox analysis and displayed using R package “forestplot.” And the survival curves were correspondingly established by Kaplan-Meier analysis to evaluate the relationship of LGG patients’ prognosis and *HMOX1* expression level as well as mutation status.

### Immune Infiltration Analysis in Lower Grade Gliomas

The Estimation of Stromal and Immune cells in Malignant Tumor tissues using Expression data (ESTIMATE; R package “estimate”) analysis was employed to measure the tumor purity. Meanwhile, we used the CIBERSORT and the Tumor Immune Estimation Resource (TIMER) algorithm to assess the fractions of human immune infiltrating cell types (Li et al., 2017; Chen et al., 2018). The abundance of these cells was compared between groups with either high or low *HMOX1* expression levels (according to the median value) in WHO grade II/III gliomas. Besides, the correlations between *HMOX1* and tumor stemness, tumor mutational burden, as well as microsatellite instability (MSI) were assessed based on Spearman’s correlation analysis, which were displayed as radar charts using R package “radar.”

### Immunohistochemical Staining and RNA Sequencing of Glioma Samples

We included 29 glioma samples from the Department of Neurosurgery, Xiangya Hospital from February 2019 to February 2021. Patients with recurrent gliomas, glioblastomas, or other cancers, or serious underlying diseases were excluded. Five fresh glioma samples were collected and then immediately stored in 4% paraformaldehyde in room temperature. Slides were sequentially incubated in graded ethanol after deparaffinization for 3 h at 60 degrees. Antigen was exposed using citrate buffer (pH = 6.0). After blocking, slides were treated with anti-*HMOX1* (rabbit, AiFang biological AF300167, 1:100) in antibody diluent (abcam ab64211), and subsequent HRP Goat anti-rabbit IgG (H+L) secondary antibody (ABclonal AS014, 1:1,000).

We acquired scanned images on immunohistochemical sections using a digital scanner, and the area of tissue measurement was automatically read using an image analysis system (Servicebio). The positive grade was divided as follows: 0 for negative staining (without staining); 1 for weakly positive (light yellow staining); 2 for moderately positive (brownish yellow staining); and 3 for strongly positive (tan staining). The histochemistry score (H-score) was calculated to reflect the degree of positivity using the following formula:


$$H-S\,c\,o\,r\,e=\sum_{i=0}^{3}\left(p_{i}\times i\right)$$

In the formula, “*p*_*i*_” indicates percentage of negative/weak/moderate/strong intensity area. “*i*” represents the positive grade. H-score ranges from 0 to 300, where a higher H-score means a stronger positivity.

Twenty-four samples were collected and then stored in liquid nitrogen for further sequencing on a BGISEQ-500 platform (BGI-Shenzhen, China). The gene expression levels were calculated using RSEM (v1.2.12). This study was approved by the Ethics Committee of Xiangya Hospital (No. 2017121019). The written informed consents were obtained in advance from all participants or their family representatives.

### Construction and Validation of Risk Signature Based on *HMOX1*-Related Genes

We calculated the correlations between *HMOX1* and other genes to identify HRGs (| correlation coefficient| > 0.6; false discovery rate (FDR) < 0.001), which were recorded for subsequent functional enrichment analysis. Gene Ontology (GO) and Kyoto Encyclopedia of Genes and Genomes (KEGG) terms were used to explore the biological functions of the gene set. Next, we developed a HRGs based prognostic signature for the LGG patients by performing the least absolute shrinkage and selection operator (LASSO) Cox regression analysis based on the R package “glmnet” (Friedman et al., 2020), The risk score calculating formula is:


$$R\,i\,s\,k\,s\,c\,o\,r\,e=\sum_{i=1}^{n}\left(\beta_{i}\times x_{i}\right)$$

Where “*n*” means the number of genes, “*β*_*i*_” is the coefficient for each gene, “*x*_*i*_’’ means the expression value (log transformed) of each gene. The predicted protein-protein interactions (PPI) among these model genes were achieved by GeneMANIA.^1^ And their correlations were also calculated.

We then calculated the risk score for the patients, and divided them into low- and high-risk groups according to the median risk score. The relationships between risk signature and survival as well as other clinicopathological characteristics were also assessed.

### Prediction of Therapy Efficacy, Correlation With Pathways in Single Cell Landscape, and Drug Response

The efficacies of four therapies (radiotherapy, chemotherapy, targeted therapy, and immunotherapy) in high-risk and low-risk groups were evaluated.

The Tumor immune dysfunction and exclusion (TIDE) was used to assess the response of LGG patients to the immunotherapies (Jiang et al., 2018). Three resources for therapeutic biomarker discovery in cancer cells, including Genomics of Drug Sensitivity in Cancer (GDSC), Cancer Therapeutics Response Portal (CTRP), and Cancer Cell Line Encyclopedia (CCLE), were employed to evaluate the relationship between drug sensitivity [half maximal inhibitory concentration (IC50)] and mRNA expression of genes.

Single-cell RNA sequencing data derived from the single-cell dataset GSE84465 which contains four cell groups and 3,590 cells (2,342 tumor cells and 1,248 periphery cells) (Darmanis et al., 2017). The correlations between gene expression and functional states including angiogenesis, apoptosis, cell cycle, differentiation, DNA damage, DNA repair, epithelial-mesenchymal transition (EMT), hypoxia, inflammation, invasion, metastasis, proliferation, quiescence, and stemness, were calculated based on the CancerSEA database^2^ (Yuan et al., 2019).

### Establishment of Nomogram

We constructed a nomogram integrating clinical information and prognostic signature by R package “rms” using the variables screened out by Cox regression analysis, which could predict the 1-, 3-, and 5-year overall survival of LGG patients in an intuitionistic and easy-to-utilize manner. Calibration plots and C-indexes were obtained to access the predictive accuracy of the model.

### Statistical Analyses

We used Kaplan-Meier curve combined log-rank test to evaluate the patient survival. Subgroups were stratified on the basis of clinical, pathological, and molecular features including age (≤ 40 or > 40-year-old), gender (male or female), grade (II or III), histological type (astrocytoma, oligoastrocytoma, or oligodendroglioma), and *IDH1* status (mutant or wildtype). When comparing variables between groups, we used Wilcoxon test or Kruskal-Wallis test. Most statistical analyses were achieved via R language (version 4.0.3). And *P* < 0.05 was considered statistically significant.

## Results

### Heme Oxygenase-1 Is Overexpressed in Lower Grade Glioma

**FIGURE 1:**
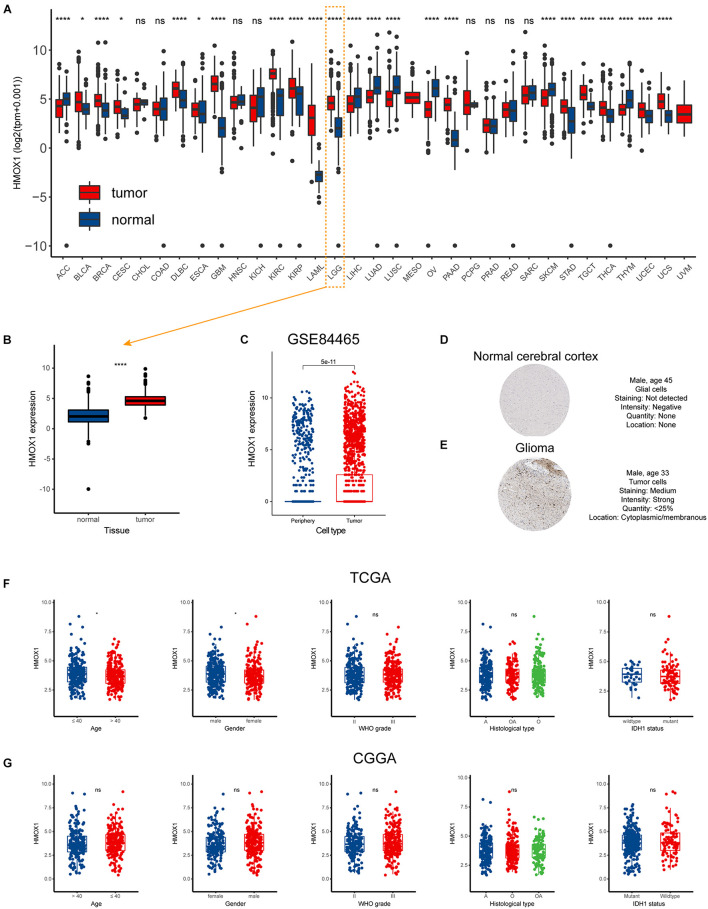
Expression profiles of *HMOX1* in human normal and cancer tissues. **(A)** *HMOX1* expression levels in TCGA pan-cancers. **(B)** *HMOX1* mRNA expression levels in LGG and normal brain tissues. **(C)** *HMOX1* mRNA expression levels in tumor and periphery single cells in GSE84465 cohort. **(D,E)** Representative IHC images of HMOX1 protein expression in normal cerebral cortex and glioma. **(F,G)** The *HMOX1* expression of different subgroups for age, gender, WHO grade, histological type, and IDH1 status in the TCGA and CGGA cohorts. *p < 0.05, ****p < 0.0001, ns, not significant. A, astrocytoma; O, oligodendroglioma; OA, oligoastrocytoma; WHO, World Health Organization; IDH1, isocitrate dehydrogenase 1.

The flow chart of our study was displayed as Supplementary Figure 1. And the characteristics of included patients were summarized in Table 1. *HMOX1* expression levels were significantly elevated in tumor samples compared to normal controls (Figures 1A,B). Importantly, this trend remained consistent at single-cell level (Figure 1C). Similarly, glioma tissue had elevated protein level of HMOX1 than normal cerebral cortex tissue (Figures 1D,E). In the TCGA cohort, youngers (≤40 years old) and males had relatively higher *HMOX1* expression levels (Figure 1F), while no significant difference was observed in CGGA cohort (Figure 1G), which could be partially attributed to the heterogeneity between cohorts.

**TABLE 1:** The characteristics of include cohorts.

**Characteristics**	**TCGA (*n* = 508)**		**CGGA (*n* = 444)**		**Samples (*n* = 29)**	
	**N**	**%**	**N**	**%**	**N**	**%**
**Age (y)**						
≤40	251	49.41	229	51.58	6	20.69
>40	257	50.59	214	48.20	23	79.31
NA	0	0.00	1	0.23	0	0.00
**Gender**						
Female	282	55.51	193	43.47	6	20.69
Male	226	44.49	251	56.53	23	79.31
**Grade**						
II	246	48.43	189	42.57	12	41.38
III	262	51.57	255	57.43	17	58.62
**Histological type**						
A	128	25.20	271	61.04	9	31.03
OA	188	37.01	30	6.76	0	0.00
O	192	37.80	142	31.98	3	10.34
NA	0	0.00	1	0.23	17	58.62
***IDH1* status**						
Wildtype	34	6.69	96	21.62	6	20.69
Mutation	91	17.91	307	69.14	18	62.07
NA	383	75.39	41	9.23	5	17.24
**Radiation therapy**						
Yes	142	27.95	315	70.95	0	0.00
No	119	23.43	102	22.97	0	0.00
NA	247	48.62	27	6.08	29	100.00
**Chemotherapy**						
Yes	277	54.53	285	64.19	0	0.00
No	229	45.08	132	29.73	0	0.00
NA	2	0.39	27	6.08	29	100.00

*A, astrocytoma; IDH1, isocitrate dehydrogenase 1; NA, not available; O, oligodendroglioma; OA, oligoastrocytoma.*

Furthermore, we comprehensively investigated the expression distribution of HMOX1 at tissue and cell levels in HPA database (Supplementary Figure 2). In normal tissues, HMOX1 was enriched in lymphoid tissues, especially spleen (Supplementary Figure 2A). Regarding cell types, HMOX1 demonstrated a high expression level in blood and immune cells including monocytes, macrophages, Kupffer cells, and Hofbauer cells (Supplementary Figure 2B). And in both tumor tissues and cell lines, HMOX1 was found to be elevated in glioma tissues and cells (U-138 MG and U-87 MG; Supplementary Figures 2C,D).

Moreover, the mutational profiles of *HMOX1* have been illustrated in Supplementary Figure 3. Amplification stood as the most common mutation type in LGG cohort (Supplementary Figure 3A). R123H/C site alteration was frequently observed among *HMOX1* mutations (Supplementary Figure 3B). Compared to the *HMOX1*-wildtpe group, *HMOX1*-mutant group shared significantly higher mutation frequencies of *TP53*, *TTN*, *MCM5* in pan-cancers (Supplementary Figure 3C), and higher mutation frequencies of *MCM5*, *APOL6*, *MB* in lower grade gliomas (Supplementary Figure 3D).

### Heme Oxygenase-1 Predicts Poorer Survival Outcome in Lower Grade Glioma

**FIGURE 2:**
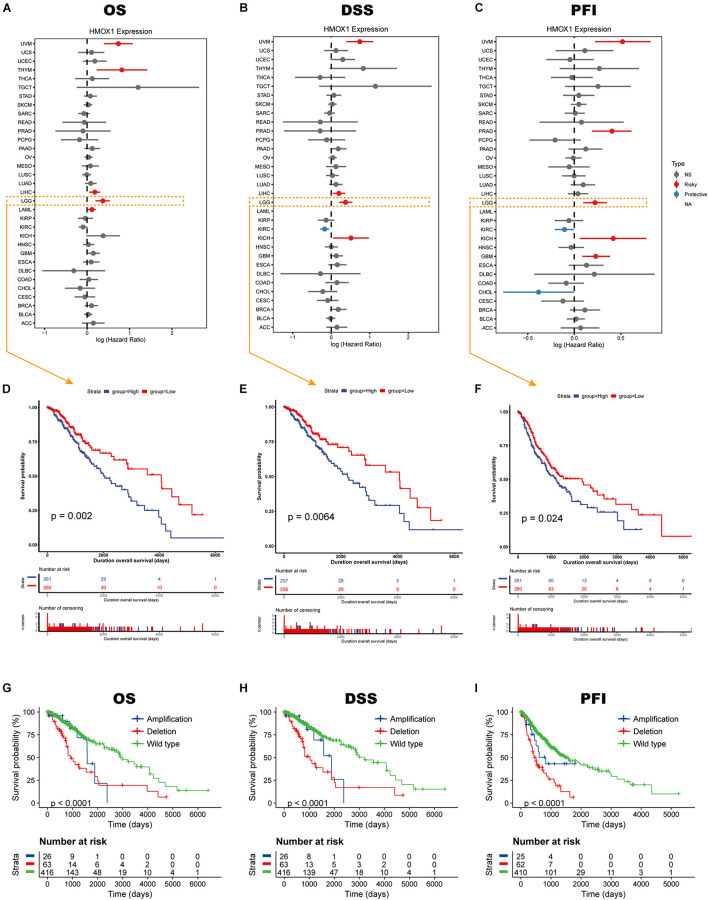
Survival profiles of *HMOX1* in cancers. Relationships between *HMOX1* expression and **(A)** OS, **(B)** DSS, **(C)** PFI across 33 cancer types in TCGA database. **(D–F)** Kaplan-Meier survival curves based on *HMOX1* expression. **(G–I)** Kaplan-Meier survival curves based on *HMOX1* CNV status.

Elevated *HMOX1* expression was significantly correlated with an unfavorable OS, DSS, and PFI in both uveal melanoma (UVM) and LGG (Figures 2A–C). Subsequent Kaplan-Meier survival analysis showed that *HMOX1*^high^ patients had significantly poorer OS (*p* = 0.0020), poorer DSS (*p* = 0.0064), and poorer PFI (*p* = 0.0240) in TCGA cohort (Figures 2D–F). Furthermore, LGG patients with *HMOX1* wildtype had better survival than those with *HMOX1* mutation (Figures 2G–I). Specifically, patients with *HMOX1* CNV deletion demonstrated the worst prognosis which can be explained by the fact that lower CNV (deletion when < 0) represented higher *HMOX1* gene expression, as CNV was negatively correlated with expression for *HMOX1* (Supplementary Figures 4A,B). Notably, when exploring the impact of the CNV status on the prognosis of glioma patients, the sample sizes varied considerably. Briefly, there were 416 individuals in the HMOX1 “wild type” group vs. only 26 in the “amplification” group. This between-group variation is highly likely to contribute to a misinterpretation of statistical results.

### Heme Oxygenase-1 Participates in Immune Infiltration and Tumor Microenvironment

**FIGURE 3:**
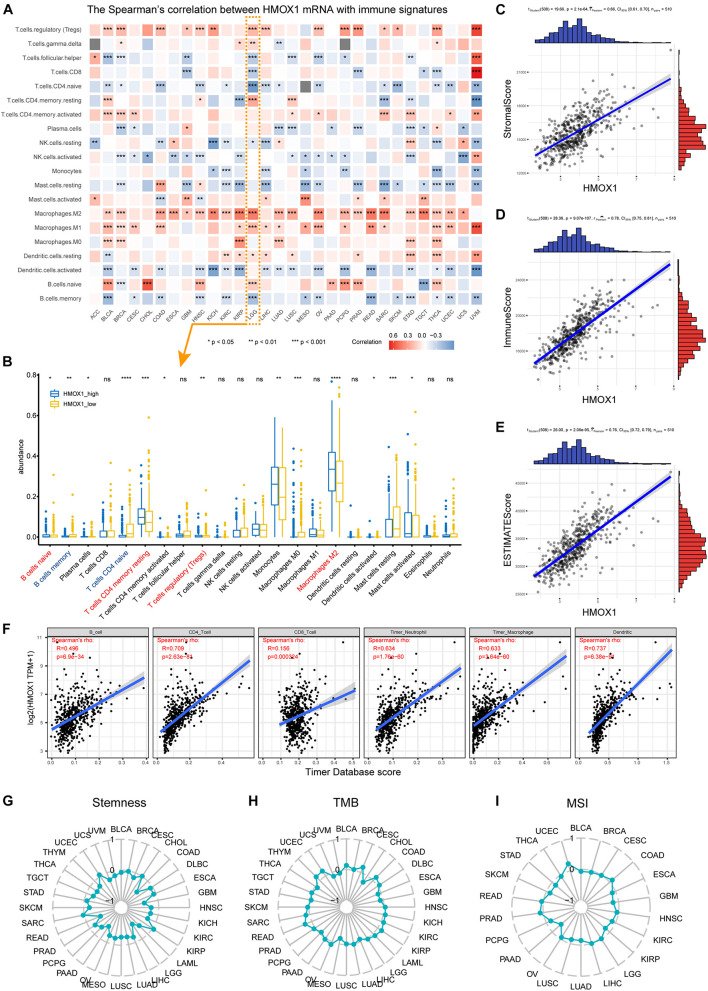
Immune infiltration profiles of *HMOX1*. **(A)** Correlations between *HMOX1* expression and immune infiltrates in pan-cancers. **(B)** The abundance of immune infiltrates between *HMOX1*^high^ and *HMOX1*^low^ groups in LGG cohort based on CIBERSORT algorithm. **(C–E)** Correlations between *HMOX1* expression and stromal, immune, ESTIMATE scores. **(F)** The correlations between *HMOX1* expression and immune infiltrates in LGG cohort based on TIMER algorithm. **(G–I)** Correlations between *HMOX1* expression and stemness indexes, TMB, MSI in pan-cancers. *p < 0.05, **p < 0.01, ***p < 0.001. TMB, tumor mutation burden; MSI, microsatellite instability.

Positively correlations were observed between *HMOX1* expression and regulatory T cells (Tregs), activated CD4+ memory T cells, as well as M2 macrophages (Figure 3A) in most cancer types. Regarding LGG, significant correlations were observed between higher *HMOX1* expression with increased abundance of naïve B cells, resting CD4+ memory T cells, Tregs, and M2 macrophages, but with decreased abundance of memory B cells and naive CD4+ T cells (Figure 3B). Furthermore, a negative relationship existed between *HMOX1* expression and tumor purity in lower grade gliomas, as it positively correlated with stromal, immune, and ESTIMATE scores (Figures 3C–E). Moreover, we found that the abundances of CD4+ T cells, neutrophils, macrophages, and dendritic cells were positively associated with *HMOX1* expression based on TIMER algorithm (Figure 3F).

To further explore the specific correlations between *HMOX1* and subpopulations of M2 macrophages. We summarized the functions, inducers, and surface markers of M2a∼d macrophages (Colin et al., 2014; Martinez and Gordon, 2014; Wang et al., 2019). Then we calculated the correlation between *HMOX1* and each marker to reflect the correlations between *HMOX1* mRNA expression and subclassified M2 macrophages (Table 2). The results showed that *HMOX1* expression was strongly correlated with M2b macrophages, as it was significantly correlated with M2b markers including CD86 (*R* = 0.752, *p* < 0.0001; Supplementary Figure 4C), HLA-DR (*R* = 0.705, *p* < 0.0001; Supplementary Figure 4D), HLA-DP (*R* = 0.672, *p* < 0.0001; Supplementary Figure 4E), and HLA-DQ (*R* = 0.621, *p* < 0.0001; Supplementary Figure 4F).

**TABLE 2:** The correlations between *HMOX1* and M2 macrophage phenotypes in LGG based on cell markers.

**M2 Macrophage phenotype**	**Functions**	**Inducers**	**Markers and surface molecules**	**Gene name**	**Spearman’s rho**	***p*-value**
M2a macrophage	Type II inflammation;	IL-4	CD163	*CD163*	0.584	1.49E-48
	Allergy;	IL-13	CD200R1	*CD200R1*	0.279	1.15E-10
	Killing and encapsulation of parasites;		CD301	*CLEC10A*	0.513	4.70E-36
	Anti-inflammation;		CXCR1	*CXCR1*	0.161	2.37E-04
	Wound healing.		CXCR2	*CXCR2*	0.526	4.28E-38
			CD209	*CD209*	0.267	6.65E-10
			Dectin-1	*CLEC7A*	**0.649** ^a^	4.28E-63
			FCERA	*FCER1A*	0.441	6.18E-26
			IL-1R2	*IL1R2*	0.280	9.59E-11
			IL-4R	*IL4R*	0.589	2.03E-49
			CD206	*MRC1*	0.090	0.0409

M2b macrophage	Th2 activation;	Lipopolysaccharides	CD86	*CD86*	**0.752** ^a^	5.48E-59
	Immunoregulation;	Immune complexes	HLA-DR	*HLA-DRA*	**0.705** ^a^	1.30E-78
	Promoting infection;	IL1R/TLR ligands	HLA-DP	*HLA-DPA1*	**0.672** ^a^	5.00E-69
	Tumor progression.		HLA-DQ	*HLA-DQA1*	**0.621** ^a^	2.70E-56
			IL-4R	*IL4R*	0.589	2.03E-49
			CD206	*MRC1*	0.090	0.0409

M2c macrophage	Immunosuppression;	IL-10	CCR2	*CCR2*	0.506	6.69E-35
	Tissue remodeling;	TGF-β	CD150	*SLAMF1*	0.427	2.86E-24
	Phagocytosis.	Glucocorticoid	CD163	*CD163*	0.584	1.49E-48
			IL-4R	*IL4R*	0.589	2.03E-49
			CD206	*MRC1*	0.090	0.0409
			SR-AI	*MSR1*	**0.758** ^a^	1.72E-97
			TLR1	*TLR1*	**0.685** ^a^	8.93E-73

M2d macrophage	Tumor progression;	TLR+A2R ligands	iNOS	*NOS2*	–0.232	9.24E-08
	Angiogenesis.		TNF-α (low)	*TNF*	0.310	5.67E-13
			IL-12 (low)	*IL12A*	0.089	0.0440
			VEGF	*VEGFA*	0.095	0.0305

***^a^**Rho greater than 0.6 was bold.*

*CXCR1, C-X-C chemokine receptor type 1; CXCR2, C-X-C chemokine receptor type 2; CLEC7A, C-type lectin domain family 7, member A; FCER1A, Fc fragment of IgE; MRC1, Mannose receptor, C type 1; CCR2, Chemokine (C-C motif) receptor 2; SLAMF1, Signaling lymphocytic activation molecule family member 1; MSR1, Macrophage scavenger receptor 1; TLR1, Toll-like receptor 1; NOS2, Nitric oxide synthase 2, inducible; VEGFA, Vascular endothelial growth factor A.*

**FIGURE 4:**
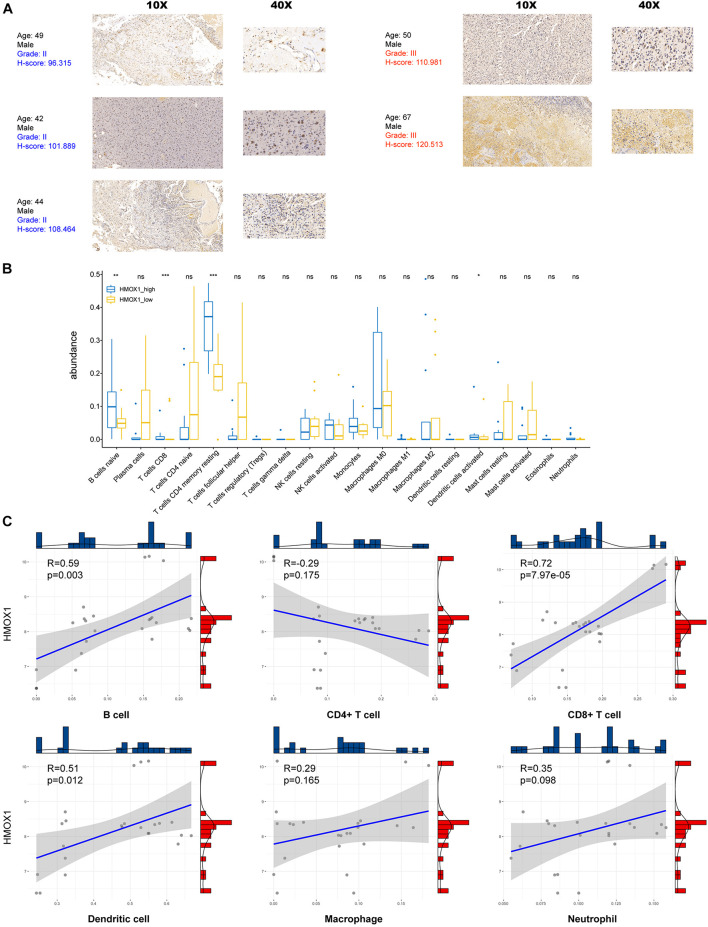
Validation by using glioma samples from Xiangya Hospital. **(A)** Immunohistochemical staining results of five samples in Xiangya hospital. **(B)** The abundance of immune infiltrates between *HMOX1*^high^ and *HMOX1*^low^ groups in 24 glioma samples. **(C)** The correlations between *HMOX1* expression and immune infiltrates in 24 glioma samples. *p < 0.05, **p < 0.01, ***p < 0.001, ns, not significant.

Using our own samples, we validated that grade III gliomas had higher HMOX1 protein expression compared with grade II gliomas (Figure 4A). The detailed immunohistochemical staining results were summarized in Supplementary Table 2. Furthermore, we confirmed the positive relationships between *HMOX1* expression levels and immune infiltrates including B cell and resting memory CD4+ T cell (Figures 4B,C), which was in consistent with the results obtained from the TCGA cohort. However, the poor correlations between *HMOX1* expression and cancer stemness, TMB, and MSI (Figures 3G–I) indicated that *HMOX1* was unlikely to influence oncogenic processes by engaging in genetic alterations or epigenetic modifications. Furthermore, *HMOX1* was significantly correlated with several recognized immune checkpoints including leukocyte associated immunoglobulin like receptor 1 (LAIR1), CD80, programmed cell death 1 ligand 2 (PDCD1LG2), CD40, and CD86 (Supplementary Figure 4G).

### Risk Signature Construction in the Training Set

**FIGURE 5:**
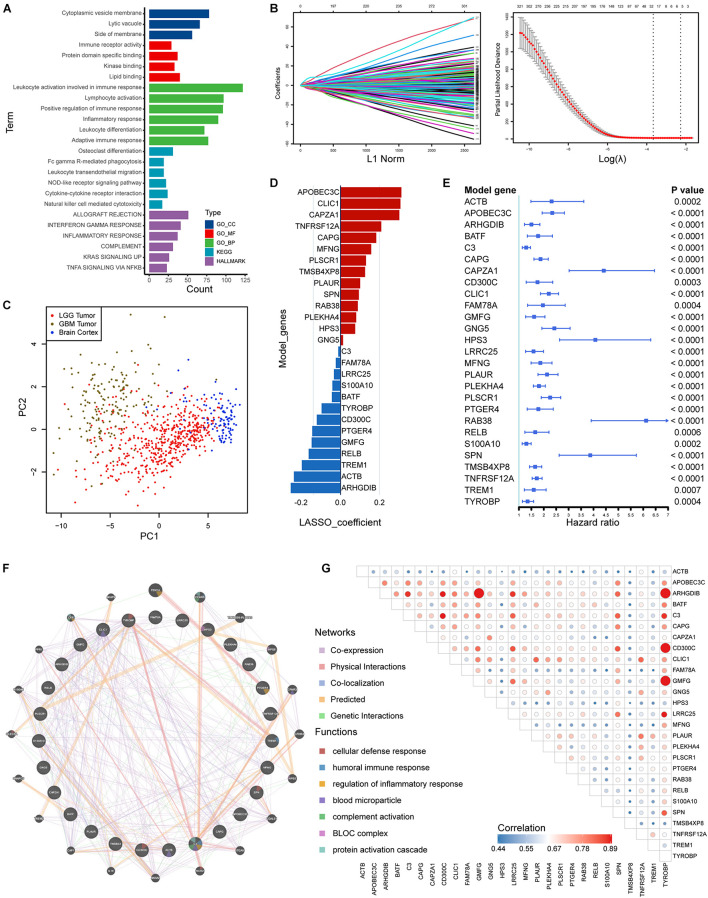
Construction of risk signature based on HRGs in the training set. **(A)** Enrichment analyses based on HRGs. **(B,C)** LASSO analysis with eligible lambda value. **(C)** PCA analysis assessing the signature. **(D)** LASSO coefficients of the model genes. **(E)** Univariate analysis results of the model genes. **(F)** Protein-protein interactions of products of included genes. **(G)** Correlations among the model genes. GO, Gene Ontology; KEGG, Kyoto Encyclopedia of Genes and Genomes.

We identified 505 HRGs (| Spearman R| > 0.6, FDR < 0.001). Mainly located on cytoplasmic vesicle membrane, HRGs participated molecular functions such as immune receptor activity and interactions. In terms of biological processes, the gene set was mainly involved in leukocyte activity involved in immune response, leukocyte activation, positive regulation of immune response, etc. (Figure 5A). Regarding KEGG pathways, HRGs were intimately involved in tumor immunity such as NOD-like receptor signaling pathway, cytokine-cytokine receptor interaction, and NK cell mediated cytotoxicity (Figure 5A).

A total of 377 HRGs were significantly prognostic in the training set following a univariate Cox analysis (*p* < 0.05). A LASSO Cox analysis was performed on training dataset and a risk signature was then constructed containing 27 genes (Figure 5B). The result of PCA analysis indicated that the signature could discriminate lower grade gliomas from GBM and normal brain tissues (Figure 5C). Moreover, the coefficients and univariate Cox analysis results were displayed in Figures 5D,E and Supplementary Table 3. Protein-protein interactions and transcriptomic correlations were also illustrated in Figures 5F,G.

### External and Subgroup Validation Demonstrates Stability of the Risk Signature

**FIGURE 6:**
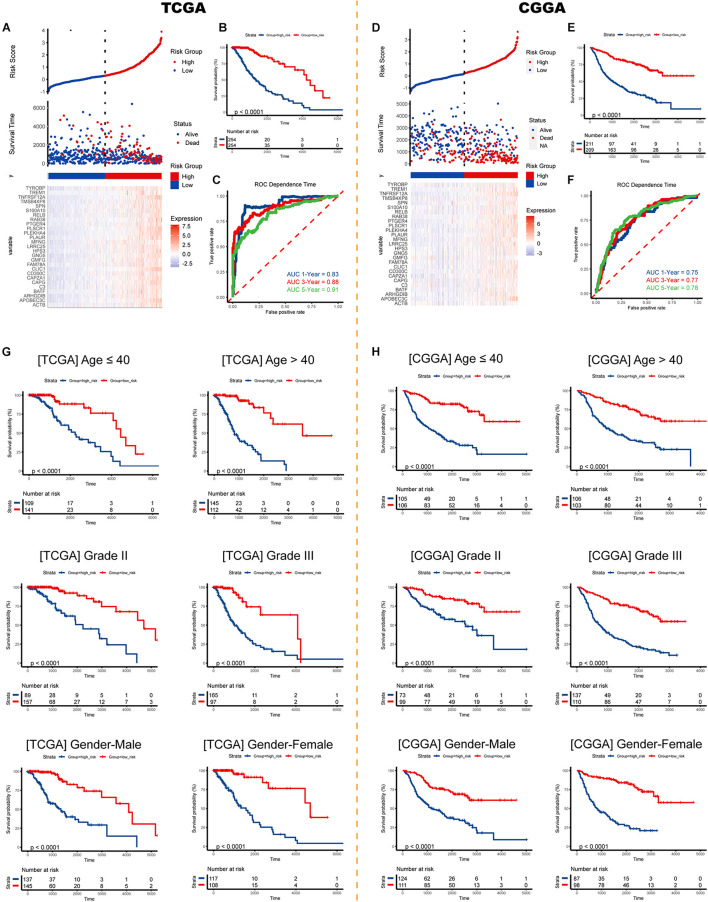
Validation of the risk signature. Risk score, survival status, and expression pattern of model genes in the **(A)** TCGA cohort and **(D)** CGGA cohort. Kaplan-Meier analysis of patients in the high- and low-risk groups in the **(B)** TCGA cohort and **(E)** CGGA cohort. Time-dependent ROC analysis of risk score in predicting prognoses in the TCGA cohort **(C)** and the CGGA cohort **(F)**. Survival analyses in subgroups in the **(G)** TCGA cohort and **(H)** CGGA cohort. ROC, receiver operating characteristic.

Risk plots and survival diagrams were displayed as Figure 6A. Kaplan-Meier curve indicated that high-risk LGG patients had significantly poorer prognosis (*p* < 0.0001, Figure 6B). The time-dependent ROC curves exhibited a promising ability of the model to predict OS in the training cohort (1-year AUC = 0.83, 3-year AUC = 0.88, 5-year AUC = 0.91; Figure 6C). The results were similar in the external CGGA cohort (Figure 6D). Higher risk scores also indicated poorer OS (*p* < 0.0001, Figure 6E). The risk model still exhibited stable and high predication ability (1-year AUC = 0.75, 3-year AUC = 0.77, 5-year AUC = 0.78; Figure 6F). Furthermore, we performed a stratification analysis and found that the risk model retained the ability to predict OS in various subgroups in both cohorts (Figures 6G,H).

### Risk Signature Correlates With Clinicopathological Characteristics, Immune Microenvironment, and Therapeutic Efficacy

**FIGURE 7:**
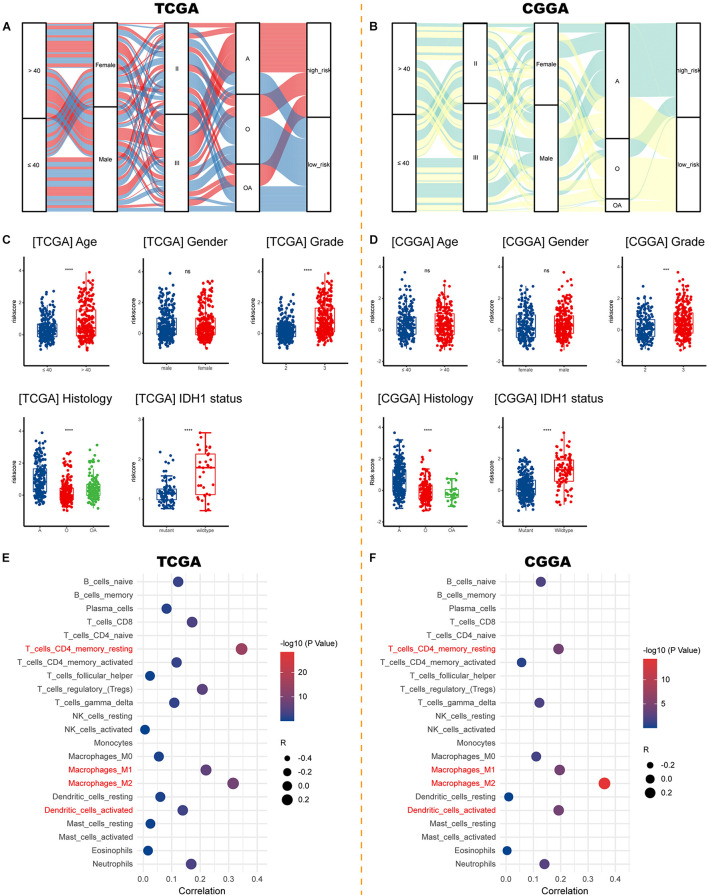
Correlations between risk signature and clinicopathological characteristics. **(A,B)** Sankey diagrams showing the distribution of risk score and clinicopathologic characteristics in the TCGA and CGGA cohorts. **(C,D)** The risk score in different subgroups stratified by age, gender, WHO grade, histological type, and IDH1 status in the TCGA and CGGA cohorts. **(E,F)** Correlations between risk score and immune infiltration in the TCGA and CGGA cohorts. ***p < 0.001, ****p < 0.0001, ns, not significant. A, astrocytoma; O, oligodendroglioma; OA, oligoastrocytoma; IDH1, isocitrate dehydrogenase 1.

Sankey diagrams were displayed showing the distribution of risk score and clinicopathologic characteristics among LGG patients (Figures 7A,B). LGG patients with increasing age and higher WHO grade possessed higher risk scores. Besides, an individual patient would have a higher risk score if his pathologic type of glioma was an astrocytoma or if he carried a wildtype IDH1 (Figures 7C,D). As for the immune microenvironment of glioma, samples with higher risk demonstrated higher percentages of resting CD4+ memory T cells, M1, M2 macrophages, and activated dendritic cells in both cohorts (Figures 7E,F and Supplementary Table 4).

**FIGURE 8:**
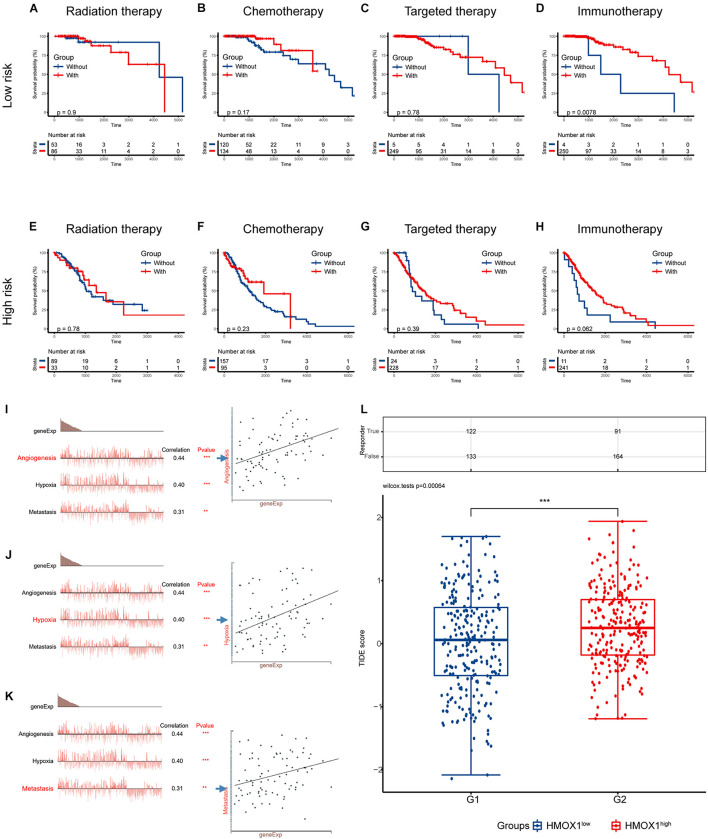
Validation using collected samples and prediction of therapy efficacy. **(A–D)** The efficacies of radiation therapy, chemotherapy, targeted therapy, and immunotherapy in the TCGA cohort. **(E–H)** The efficacies of radiation therapy, chemotherapy, targeted therapy, and immunotherapy in the CGGA cohort. **(I–K)** The relationship between functional states and *HMOX1* expression based on single-cell sequencing. **(L)** Prediction of response to immunotherapy using TIDE algorithm. **p < 0.01, ***p < 0.001. TIDE, Tumor Immune Dysfunction and Exclusion.

As for the association between risk signature and therapeutic benefit, most therapies showed poor efficacy in both low-risk (Figures 8A–C) and high-risk groups (Figures 8E–G) in the TCGA cohort. However, patients who had lower risk scores exhibited uniquely better prognosis if they had undergone immunotherapies (*p* = 0.0078; Figure 8D). But the condition was not observed in the high-risk group (*p* = 0.0620; Figure 8H).

We then decoded the different functional states of malignant cells and target molecules at single-cell resolution and discovered three functional states which were significantly related to *HMOX1*, including angiogenesis (correlation = 0.44, *p* < 0.001; Figure 8I), hypoxia (correlation = 0.40, *p* < 0.001; Figure 8J), and metastasis (correlation = 0.31, *p* < 0.01; Figure 8K). Furthermore, patients with lower *HMOX1* expression levels had high TIDE scores and exhibited better responses to the immunotherapies (Figure 8L). This is in consistent with the results of prediction of therapy efficacy in different risk subgroups. In addition, three drug databases were accessed to find relationship between drug sensitivity and *HMOX1* expression. TKI258 (dovitinib) showed significant sensitivity in the CCLE database (Supplementary Figure 5A). WZ3105 and Repligen 136 were selected from the CTRP (Supplementary Figure 5B) and GDSC (Supplementary Figure 5C) drug banks, respectively.

### Construction and Validation of the Nomogram

**FIGURE 9:**
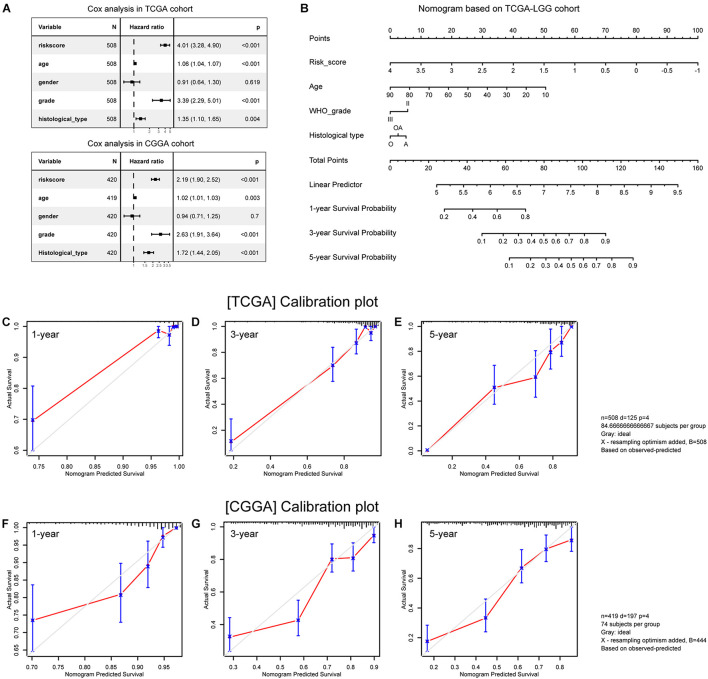
Construction and validation of the nomogram. **(A)** Multivariate analysis results. **(B)** Nomogram based on risk score, age, WHO grade, and histological type. **(C–E)** Calibration plots of the nomogram for predicting the probability of OS at 1-, 3-, and 5-year in the TCGA cohort. **(F–H)** Calibration plots of the nomogram for predicting the probability of OS at 1-, 3-, and 5-year in the CGGA cohort. A, astrocytoma; O, oligodendroglioma; OA, oligoastrocytoma.

Multivariate Cox analyses showed that age at first pathological diagnosis, WHO grade, histological type, and risk score were independent predictors of OS in both cohorts (Figure 9A). We then constructed a nomogram model incorporating these prognostic variables as a clinically applicable quantitative tool to predict survival in patients with primary lower grade gliomas (Figure 9B). The calibration plots demonstrated satisfactory predictive performance of the nomogram in both training (Figures 9C–E) and validation (Figures 9F–H) cohorts.

## Discussion

Molecular profiling of lower grade gliomas, common tumors in the human brain, has enhanced insights of molecular oncology and identified few prognostic and therapeutic targets. Over the past decades, surgical section following radiotherapy and chemotherapy still serve as the mainstream means for glioma treatment. Due to the unique microenvironment and low mutational burden, gliomas acquire immunosuppressive phenotypes and poorly response to established immunotherapies. Clinicians thus need renewed molecular targets for better response rate as well as improved prognosis. Comprehensive bioinformatic analyses were used at multiple levels of expression, survival, and biological function to demonstrate the essential role of *HMOX1*. *HMOX1* and the risk signature are reliable predictors of LGG prognosis, and targeting *HMOX1* will help clinicians optimize the management of lower grade glioma patients in future preclinical and clinical practice.

The mechanism can be explained by the antioxidant role of HMOX1 in malignancies. Heme oxygenase-1 is a phase II enzyme that responds to adverse conditions, such as oxidative stress, cellular injury, and diseases. Its diverse roles in tumorigenesis have been well reviewed (Nitti et al., 2017; Chiang et al., 2018). Furthermore, HMOX1 has been widely recognized to play a cytoprotective role in tumor cells to overcome the assault of enhanced oxidative stress in the tumor microenvironment, thereby preventing the cancer cells from apoptosis and autophagy (Chiang et al., 2018). On the other hand, our study found that HMOX1 was enriched in blood and immune cells, especially monocytes and macrophages, which were thought to be risk factors for the overall survival of glioma patients (Zhang et al., 2021a,b). Therefore, a higher *HMOX1* expression level represented a stronger resistance to oxidative stress and a higher abundance of risk immune cell types, resulting an unfavorable outcome for LGG patients.

We observed that *HMOX1* expression was increased in lower grade gliomas at both tissue and single-cell levels, and such increase was associated with worse OS, DSS, and PFI in patients with LGG. Thereafter we suspect that *HMOX1* might influence the malignant properties and patient outcomes by intervening immune infiltration in the TME, as it was significantly associated with tumor purity, immune checkpoints’ levels, immune infiltration abundance, as well as immune-oncological pathways. These findings were partially validated by our own samples.

In this study, 27of 377 prognostically valuable HRGs were screened out to establish a risk signature. Among them, *ARHGDIB* (Su et al., 2019), *LRRC25* (Zhang et al., 2020), *PLAUR* (Tan et al., 2020), and *TREM1* (Kong et al., 2020) were selected as prognostic hub genes in lower grade glioma in previous bioinformatic analyses. Besides, *GMFG* (Lan et al., 2021), *GNG5* (Yang et al., 2020), *S100A10* (Zhang et al., 2021c), *TNFRSF12A* (Tran et al., 2006), and *TYROBP* (Lu et al., 2021) were found to be upregulated and correlated with worse prognosis in LGG. Importantly, *CAPG* was identified as a prognosis factor correlated with macrophages in gliomas (Wei et al., 2020; Prescher et al., 2021). Moreover, a recent study reported that targeting *CLIC1* reduced gliomagenesis in tumoral stem or progenitor cells, indicating *CLIC1* as a potential target and prognostic biomarker there (Wang et al., 2012; Setti et al., 2013). Briefly, previous literature indicated that the model genes and encoded proteins were related to the progression of gliomas, which were perfectly consistent to our risk signature. The risk signature we constructed may reflect the distribution of immune infiltrates involved in the TME, as well as reliably and independently predicts prognosis of patients with primary gliomas. In addition, combining the constructed signature with clinicopathological features, we obtained a nomogram with strong predictive power which is of potential in clinical utilization.

Intriguingly, both the risk signature and *HMOX1* expression are correlated with fractions of infiltrating cells, especially resting memory CD4+ T cells and M2 macrophages. Resting memory CD4+ T cells were indicated to play an vital role in latent HIV-1 infection (Siliciano and Siliciano, 2015), its functions is yet warranted to be discovered in glioma. Additionally, abundance of M2 macrophages was associated with immune suppressive phenotype and short-term relapse after radiation therapy (Wang et al., 2017). Moreover, M2b macrophages are the most closely related to *HMOX1* in M2 macrophage subsets. Considering its involvement in type 2 helper T (Th2) activation, immunomodulation, and tumor progression, combined with the fact that Th2/Th1 ratio is a poor prognostic marker in gliomas (Kumar et al., 2006), we have good reasons to believe that HMOX1 is involved in immunoregulation in glioma microenvironment, driving tumors toward more malignant properties as well as a worse prognosis. Further work is certainly imperative for the validation of *HMOX1* functions in lower grade glioma. Besides, selectively targeting *HMOX1* was effective for therapy resistance in various cancers (Mayerhofer et al., 2008; Alaoui-Jamali et al., 2009). Several recently developed imidazole based non-porphyrin *HMOX1* inhibitors are highly selective for *HMOX1* without affecting other heme-containing proteins (Pittalà et al., 2013), and show potent antitumor activities in preclinical models (Salerno et al., 2013), exhibiting high-quality prospects for clinical use.

A key finding in our study is that immunotherapy demonstrated unique superiority in the low-risk group as patients with vs. without immunotherapy exhibited significantly better survival. Considering that patients with lower *HMOX1* expression had lower TIDE score and showed higher response rate to immunotherapy, we conclude that *HMOX1*, as well as *HMOX1*-based risk signature, is capable for predicting the immunotherapy efficacy in lower grade gliomas. Regarding the mechanisms, we found that both *HMOX1* and risk signature were correlated with M2 macrophages. An important and promising observation for cancer immunotherapy is that macrophages can direct T or B cell responses in the presence or absence of specific antigens (Mills et al., 2016). In particular, M2 macrophages induce T cells into Treg and other T cell type responses without anticancer activity via innate signals such as transforming growth factor-β (TGF-β) and interleukin 10 (IL-10) (Mills et al., 2000; Ruffell and Coussens, 2015). Therefore, we believe that the key to the sensitivity of immunotherapies to the risk score is that the *HMOX1*-based risk score well reflects the distribution of M2 macrophages in gliomas.

There were several limitations in our study. First, our sample size is too small to be completely convinced. Second, only one cohort with single-cell sequencing data was employed to evaluated the functions of *HMOX1* in glioma. Third, we included 27 genes in our risk signature, proposing a great challenge for experimental validation. Finally, The TCGA dataset does not provide details of adjuvant therapy and, in particular, immunotherapy, which reduces the generalizability of our conclusions. Unfortunately, we do not have such a LGG dataset to study the immunotherapy response. Although we used patients from the CGGA database (validation set) to validate the results obtained from the TCGA database (training set) and showed good concordance, we believe that a larger multi-center cohort of glioma patients who undergo immunotherapy will provide insight into this issue.

In our study, we employed data from independent cohorts to explore the expression profile of heme oxygenase-1 in glioma. *HMOX1* influences immune infiltration as well as survival prognosis of LGG patients. Importantly, *HMOX1* were related to oncological functional states including angiogenesis, hypoxia, and metastasis. A risk signature and nomogram based on HRGs have robust predict power as well as the potential for clinical applications.

## Data Availability Statement

The datasets presented in this study can be found in online repositories. The names of the repository/repositories and accession number(s) can be found in the article/Supplementary Material.

## Ethics Statement

The studies involving human participants were reviewed and approved by the Ethics Committee of Xiangya Hospital. The patients/participants provided their written informed consent to participate in this study.

## Author Contributions

WY performed the data analysis, interpreted the data, and prepared the draft. FL collected the samples in our cohort and they were responsible for the subsequent RNA sequencing of them. CL performed the visualization. ZL and FL revised the manuscript. CL and FL designed the research and supervised all the work. All authors have read and approved the final manuscript, and agree to be accountable for the content of the work.

## Conflict of Interest

The authors declare that the research was conducted in the absence of any commercial or financial relationships that could be construed as a potential conflict of interest.

## Publisher’s Note

All claims expressed in this article are solely those of the authors and do not necessarily represent those of their affiliated organizations, or those of the publisher, the editors and the reviewers. Any product that may be evaluated in this article, or claim that may be made by its manufacturer, is not guaranteed or endorsed by the publisher.

## Footnotes

1
http://genemania.org/

2
http://biocc.hrbmu.edu.cn/CancerSEA

## Supplementary Material

Supplementary Figure 1 Flow chart of the whole study. FDR, false discovery rate; LASSO, least absolute shrinkage and selection operator.

Supplementary Figure 2 The comprehensive illustration of HMOX1 expression in different tissues and cell types downloading from the HPA database (https://www.proteinatlas.org/). **(A)** The expression of *HMOX1* in normal tissues. **(B)** The expression of *HMOX1* in normal cells (including single cells). **(C)** The expression of *HMOX1* in tumor tissues. **(G)** The expression of *HMOX1* in cell lines.

Supplementary Figure 3 Mutational profiles of *HMOX1*. **(A)** Summary of *HMOX1* mutations in TCGA pan-cancers. **(B)** The general mutation count of *HMOX1*. **(C,D)** Differential altered genes between HMOX1-altered and HMOX1-unaltered groups in pan-cancer and LGG cohort. **p* < 0.05. CNV, copy number variation.

Supplementary Figure 4 **(A)** CNV distribution of *HMOX1* in LGG tumor samples. **(B)** Spearman correlation between *HMOX1* CNV and mRNA expression in LGG. **(C–F)** Correlations between *HMOX1* and M2b macrophage markers. **(G)** Correlations between *HMOX1* expression and recognized immune checkpoints. ^∗^*p* < 0.05, ^∗∗^*p* < 0.01, ^∗∗∗^*p* < 0.001. CNV, copy number variation; Homo. Amp., homogenous amplification; Homo. Del., homogenous deletion; Hete. Amp., heterogenous amplification; Hete. Del., heterogenous deletion.

Supplementary Figure 5 Prediction of drug responses in the **(A)** Cancer Cell Line Encyclopedia (CCLE), **(B)** Cancer Therapeutics Response Portal (CTRP), and **(C)** Genomics of Drug Sensitivity in Cancer (GDSC) banks. **p* < 0.05.

## References

[B1] Alaoui-Jamali M. A. Bismar T. A. Gupta A. Szarek W. A. Su J. Song W. (2009). A novel experimental heme oxygenase-1–targeted therapy for hormone-refractory prostate cancer. *Cancer Res.* 69 8017–8024. 10.1158/0008-5472.CAN-09-0419 19808972

[B2] Brat D. J. Verhaak R. G. W. Aldape K. D. Yung W. K. A. Salama S. R. Cooper L. A. D. (2015). Comprehensive, integrative genomic analysis of diffuse lower-grade gliomas. *N. Engl. J. Med.* 372 2481–2498. 10.1158/1538-7445.AM2014-936 26061751 PMC4530011

[B3] Cairncross G. Wang M. Shaw E. Jenkins R. Brachman D. Buckner J. (2013). Phase III trial of chemoradiotherapy for anaplastic oligodendroglioma: long-term results of RTOG 9402. *J. Clin. Oncol.* 31 337–343. 10.1200/JCO.2012.43.2674 23071247 PMC3732012

[B4] Chen B. Khodadoust M. S. Liu C. L. Newman A. M. Alizadeh A. A. (2018). Profiling tumor infiltrating immune cells with CIBERSORT. *Methods Mol. Biol.* 1711 243–259. 10.1007/978-1-4939-7493-1_12 29344893 PMC5895181

[B5] Chiang S.-K. Chen S.-E. Chang L.-C. A. (2018). Dual role of heme oxygenase-1 in cancer cells. *Int. J. Mol. Sci.* 20:39. 10.3390/ijms20010039 30583467 PMC6337503

[B6] Colin S. Chinetti-Gbaguidi G. Staels B. (2014). Macrophage phenotypes in atherosclerosis. *Immunol. Rev.* 262 153–166. 10.1111/imr.12218 25319333

[B7] Darmanis S. Sloan S. A. Croote D. Mignardi M. Chernikova S. Samghababi P. (2017). Single-cell RNA-Seq analysis of infiltrating neoplastic cells at the migrating front of human glioblastoma. *Cell Rep.* 21 1399–1410. 10.1016/j.celrep.2017.10.030 29091775 PMC5810554

[B8] Degese M. S. Mendizabal J. E. Gandini N. A. Gutkind J. S. Molinolo A. Hewitt S. M. (2012). Expression of heme oxygenase-1 in non-small cell lung cancer (NSCLC) and its correlation with clinical data. *Lung Cancer* 77 168–175. 10.1016/j.lungcan.2012.02.016 22418244 PMC8381257

[B9] Friedman J. Hastie T. Tibshirani R. Narasimhan B. Tay K. Simon N. (2020). *Lasso and Elastic-Net Regularized Generalized Linear Models [R package glmnet version 4.0-2].*

[B10] Gandini N. A. Fermento M. E. Salomón D. G. Obiol D. J. Andrés N. C. Zenklusen J. C. (2014). Heme oxygenase-1 expression in human gliomas and its correlation with poor prognosis in patients with astrocytoma. *Tumor Biol.* 35 2803–2815. 10.1007/s13277-013-1373-z 24234335

[B11] Jiang P. Gu S. Pan D. Fu J. Sahu A. Hu X. (2018). Signatures of T cell dysfunction and exclusion predict cancer immunotherapy response. *Nat. Med.* 24 1550–1558. 10.1038/s41591-018-0136-1 30127393 PMC6487502

[B12] Kong Y. Feng Z.-C. Zhang Y.-L. Liu X.-F. Ma Y. Zhao Z.-M. (2020). Identification of immune-related genes contributing to the development of glioblastoma using weighted gene co-expression network analysis. *Front. Immunol.* 11:1281. 10.3389/fimmu.2020.01281 32765489 PMC7378359

[B13] Kumar R. Kamdar D. Madden L. Hills C. Crooks D. O’Brien D. (2006). Th1/Th2 cytokine imbalance in meningioma, anaplastic astrocytoma and glioblastoma multiforme patients. *Oncol. Rep.* 15 1513–1516. 10.3892/or.15.6.1513 16685388

[B14] Lan A. Ren C. Wang X. Tong G. Yang G. (2021). Bioinformatics and survival analysis of glia maturation factor-γ in pan-cancers. *BMC Cancer* 21:423. 10.1186/s12885-021-08163-2 33863293 PMC8052856

[B15] Li T. Fan J. Wang B. Traugh N. Chen Q. Liu J. S. (2017). TIMER: a web server for comprehensive analysis of tumor-infiltrating immune cells. *Cancer Res.* 77 e108–e110. 10.1158/0008-5472.CAN-17-0307 29092952 PMC6042652

[B16] Lu J. Peng Y. Huang R. Feng Z. Fan Y. Wang H. (2021). Elevated TYROBP expression predicts poor prognosis and high tumor immune infiltration in patients with low-grade glioma. *BMC Cancer* 21:723. 10.1186/s12885-021-08456-6 34162355 PMC8220692

[B17] Maines M. D. Abrahamsson P.-A. (1996). Expression of heme oxygenase-1 (HSP32) in human prostate: normal, hyperplastic, and tumor tissue distribution. *Urology* 47 727–733. 10.1016/S0090-4295(96)00010-6 8650873

[B18] Martinez F. O. Gordon S. (2014). The M1 and M2 paradigm of macrophage activation: time for reassessment. *F1000 Med. Rep.* 6:13. 10.12703/P6-13 24669294 PMC3944738

[B19] Mayerhofer M. Gleixner K. V. Mayerhofer J. Hoermann G. Jaeger E. Aichberger K. J. (2008). Targeting of heat shock protein 32 (Hsp32)/heme oxygenase-1 (HO-1) in leukemic cells in chronic myeloid leukemia: a novel approach to overcome resistance against imatinib. *Blood* 111 2200–2210. 10.1182/blood-2006-11-055723 18024796

[B20] Mills C. D. Kincaid K. Alt J. M. Heilman M. J. Hill A. M. (2000). M-1/M-2 macrophages and the Th1/Th2 paradigm. *J. Immunol.* 164 6166–6173. 10.4049/jimmunol.164.12.6166 10843666

[B21] Mills C. D. Lenz L. L. Harris R. A. (2016). A breakthrough: macrophage-directed cancer immunotherapy. *Cancer Res.* 76 513–516. 10.1158/0008-5472.CAN-15-1737 26772756 PMC4738030

[B22] Miyata Y. Kanda S. Mitsunari K. Asai A. Sakai H. (2014). Heme oxygenase-1 expression is associated with tumor aggressiveness and outcomes in patients with bladder cancer: a correlation with smoking intensity. *Transl. Res.* 164 468–476. 10.1016/j.trsl.2014.06.010 25063314

[B23] Nduom E. K. Weller M. Heimberger A. B. (2015). Immunosuppressive mechanisms in glioblastoma. *Neuro Oncol.* 17 vii9–vii14. 10.1093/neuonc/nov151 26516226 PMC4625890

[B24] Nitti M. Piras S. Marinari U. M. Moretta L. Pronzato M. A. Furfaro A. L. (2017). HO-1 induction in cancer progression: a matter of cell adaptation. *Antioxidants* 6:29. 10.3390/antiox6020029 28475131 PMC5488009

[B25] Noh S. J. Bae J. S. Jamiyandorj U. Park H. S. Kwon K. S. Jung S. H. (2013). Expression of nerve growth factor and heme oxygenase-1 predict poor survival of breast carcinoma patients. *BMC Cancer* 13:516. 10.1186/1471-2407-13-516 24180625 PMC3818967

[B26] Ph L. Wm L. Ly C. (2013). TRC8 suppresses tumorigenesis through targeting heme oxygenase-1 for ubiquitination and degradation. *Oncogene* 32 2325–2334. 10.1038/onc.2012.244 22689053

[B27] Phillips A. C. Boghaert E. R. Vaidya K. S. Mitten M. J. Norvell S. Falls H. D. (2016). ABT-414, an antibody drug conjugate targeting a tumor-selective EGFR epitope. *Mol. Cancer Ther.* 15 661–669. 10.1158/1535-7163.MCT-15-0901 26846818

[B28] Pittalà V. Salerno L. Romeo G. Modica M. N. Siracusa M. A. (2013). A focus on heme oxygenase-1 (HO-1) inhibitors. *Curr. Med. Chem.* 20 3711–3732. 10.2174/0929867311320300003 23746277

[B29] Prescher N. Hänsch S. Knobbe-Thomsen C. B. Stühler K. Poschmann G. (2021). The migration behavior of human glioblastoma cells is influenced by the redox-sensitive human macrophage capping protein CAPG. *Free Radic. Biol. Med.* 167 81–93. 10.1016/j.freeradbiomed.2021.02.038 33711419

[B30] Robinson G. W. Orr B. A. Gajjar A. (2014). Complete clinical regression of a BRAF V600E-mutant pediatric glioblastoma multiforme after BRAF inhibitor therapy. *BMC Cancer* 14:258. 10.1186/1471-2407-14-258 24725538 PMC3996187

[B31] Ruffell B. Coussens L. M. (2015). Macrophages and therapeutic resistance in cancer. *Cancer Cell* 27 462–472. 10.1016/j.ccell.2015.02.015 25858805 PMC4400235

[B32] Salerno L. Pittalà V. Romeo G. Modica M. N. Siracusa M. A. Giacomo C. D. (2013). Evaluation of novel aryloxyalkyl derivatives of imidazole and 1,2,4-triazole as heme oxygenase-1 (HO-1) inhibitors and their antitumor properties. *Bioorg. Med. Chem.* 21 5145–5153. 10.1016/j.bmc.2013.06.040 23867390

[B33] Sanai N. Alvarez-Buylla A. Berger M. S. (2005). Neural stem cells and the origin of gliomas. *N. Engl. J. Med.* 353 811–822. 10.1056/NEJMra043666 16120861

[B34] Setti M. Savalli N. Osti D. Richichi C. Angelini M. Brescia P. (2013). Functional role of CLIC1 ion channel in glioblastoma-derived stem/progenitor cells. *J. Natl. Cancer Inst.* 105 1644–1655. 10.1093/jnci/djt278 24115360 PMC3818171

[B35] Siliciano J. M. Siliciano R. F. (2015). The remarkable stability of the latent reservoir for HIV-1 in resting memory CD4+ T Cells. *J. Infect. Dis.* 212 1345–1347. 10.1093/infdis/jiv219 25877551

[B36] Stefano A. L. D. Fucci A. Frattini V. Labussiere M. Mokhtari K. Zoppoli P. (2015). Detection, characterization and inhibition of FGFR-TACC fusions in IDH wild type glioma. *Clin. Cancer Res.* 21 3307–3317. 10.1158/1078-0432.CCR-14-2199 25609060 PMC4506218

[B37] Su J. Long W. Ma Q. Xiao K. Li Y. Xiao Q. (2019). Identification of a tumor microenvironment-related eight-gene signature for predicting prognosis in lower-grade gliomas. *Front. Genet.* 10:1143. 10.3389/fgene.2019.01143 31803233 PMC6872675

[B38] Suzuki H. Aoki K. Chiba K. Sato Y. Shiozawa Y. Shiraishi Y. (2015). Mutational landscape and clonal architecture in grade II and III gliomas. *Nat. Genet.* 47 458–468. 10.1038/ng.3273 25848751

[B39] Tan Y. Q. Li Y. T. Yan T. F. Xu Y. Liu B. H. Yang J. A. (2020). Six immune associated genes construct prognostic model evaluate low-grade glioma. *Front. Immunol.* 11:606164. 10.3389/fimmu.2020.606164 33408717 PMC7779629

[B40] Tran N. L. McDonough W. S. Savitch B. A. Fortin S. P. Winkles J. A. Symons M. (2006). Increased fibroblast growth factor-inducible 14 expression levels promote glioma cell invasion via Rac1 and nuclear factor-κB and correlate with poor patient outcome. *Cancer Res.* 66 9535–9542. 10.1158/0008-5472.CAN-06-0418 17018610

[B41] van den Bent M. J. Brandes A. A. Taphoorn M. J. B. Kros J. M. Kouwenhoven M. C. M. Delattre J.-Y. (2013). Adjuvant procarbazine, lomustine, and vincristine chemotherapy in newly diagnosed anaplastic oligodendroglioma: long-term follow-up of EORTC brain tumor group study 26951. *J. Clin. Oncol.* 31 344–350. 10.1200/JCO.2012.43.2229 23071237

[B42] Wang L. He S. Tu Y. Ji P. Zong J. Zhang J. (2012). Elevated expression of chloride intracellular channel 1 is correlated with poor prognosis in human gliomas. *J. Exp. Clin. Cancer Res.* 31:44. 10.1186/1756-9966-31-44 22578365 PMC3441274

[B43] Wang L. X. Zhang S. X. Wu H. J. Rong X. L. Guo J. (2019). M2b macrophage polarization and its roles in diseases. *J. Leukoc. Biol.* 106 345–358. 10.1002/JLB.3RU1018-378RR 30576000 PMC7379745

[B44] Wang Q. Hu B. Hu X. Kim H. Squatrito M. Scarpace L. (2017). Tumor evolution of glioma-intrinsic gene expression subtypes associates with immunological changes in the microenvironment. *Cancer Cell* 32:152. 10.1016/j.ccell.2017.06.003 28697342 PMC5599156

[B45] Was H. Cichon T. Smolarczyk R. Rudnicka D. Stopa M. Chevalier C. (2006). Overexpression of heme oxygenase-1 in murine melanoma: increased proliferation and viability of tumor cells, decreased survival of mice. *Am. J. Pathol.* 169 2181–2198. 10.2353/ajpath.2006.051365 17148680 PMC1762485

[B46] Wei J. Feng L. Wu L. (2020). Integrated analysis identified CAPG as a prognosis factor correlated with immune infiltrates in lower−grade glioma. *Clin. Transl. med.* 10:e51. 10.1002/ctm2.51 32536029 PMC7403817

[B47] Wick W. Platten M. Meisner C. Felsberg J. Tabatabai G. Simon M. (2012). Temozolomide chemotherapy alone versus radiotherapy alone for malignant astrocytoma in the elderly: the NOA-08 randomised, phase 3 trial. *Lancet Oncol.* 13 707–715. 10.1016/S1470-2045(12)70164-X 22578793

[B48] Yan H. Parsons D. W. Jin G. McLendon R. Rasheed B. A. Yuan W. (2009). IDH1 and IDH2 mutations in gliomas. *N. Engl. J. Med.* 360 765–773. 10.1056/NEJMoa0808710 19228619 PMC2820383

[B49] Yang B. Han Z.-Y. Wang W.-J. Ma Y.-B. Chu S.-H. (2020). GNG5 is an unfavourable independent prognostic indicator of gliomas. *J. Cell. Mol. Med.* 24 12873–12878. 10.1111/jcmm.15923 33000557 PMC7686969

[B50] Yin H. Fang J. Liao L. Maeda H. Su Q. (2014). Upregulation of heme oxygenase-1 in colorectal cancer patients with increased circulation carbon monoxide levels, potentially affects chemotherapeutic sensitivity. *Bmc Cancer* 14:436. 10.1186/1471-2407-14-436 24927633 PMC4075569

[B51] Yin Y. Liu Q. Wang B. Chen G. Xu L. Zhou H. (2012). Expression and function of heme oxygenase-1 in human gastric cancer. *Exp. Biol. Med.* 237 362–371. 10.1258/ebm.2011.011193 22490514

[B52] Yuan H. Yan M. Zhang G. Liu W. Deng C. Liao G. (2019). CancerSEA: a cancer single-cell state atlas. *Nucleic Acids Res.* 47 D900–D908. 10.1093/nar/gky939 30329142 PMC6324047

[B53] Zhang M. Wang X. Chen X. Guo F. Hong J. (2020). Prognostic value of a stemness index-associated signature in primary lower-grade glioma. *Front. Genet.* 11:441. 10.3389/fgene.2020.00441 32431729 PMC7216823

[B54] Zhang N. Dai Z. Wu W. Wang Z. Cao H. Zhang Y. (2021a). The predictive value of monocytes in immune microenvironment and prognosis of glioma patients based on machine learning. *Front. Immunol.* 12:656541. 10.3389/fimmu.2021.656541 33959130 PMC8095378

[B55] Zhang N. Zhang H. Wang Z. Dai Z. Zhang X. Cheng Q. (2021b). Immune infiltrating cells-derived risk signature based on large-scale analysis defines immune landscape and predicts immunotherapy responses in glioma tumor microenvironment. *Front. Immunol.* 12:691811. 10.3389/fimmu.2021.691811 34489938 PMC8418124

[B56] Zhang Y. Yang X. Zhu X.-L. Bai H. Wang Z.-Z. Zhang J.-J. (2021c). S100A gene family: immune-related prognostic biomarkers and therapeutic targets for low-grade glioma. *Aging (Albany NY)* 13 15459–15478. 10.18632/aging.203103 34148033 PMC8221329

