# Supplementary figures and images for: Heme Oxygenase-1 Predicts Risk Stratification and Immunotherapy Efficacy in Lower Grade Gliomas
Source: Front Cell Dev Biol. 2021 Nov 9;9:760800. doi: 10.3389/fcell.2021.760800 (PMC8631111; doi:10.3389/fcell.2021.760800)

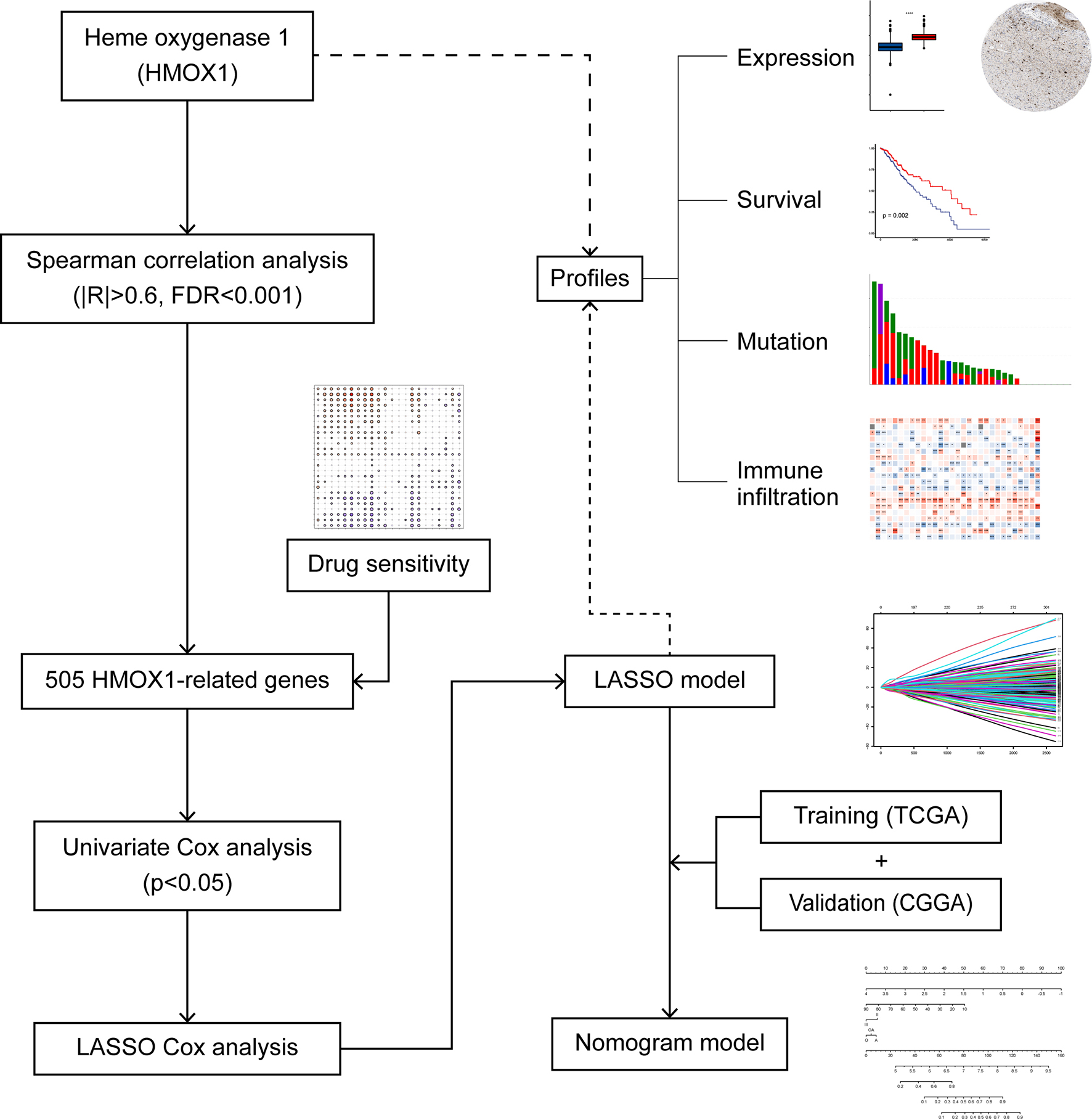

Supplement: Supplementary Figure 1 — Flow chart of the whole study. FDR, false discovery rate; LASSO, least absolute shrinkage and selection operator. [file Image_1.JPEG]

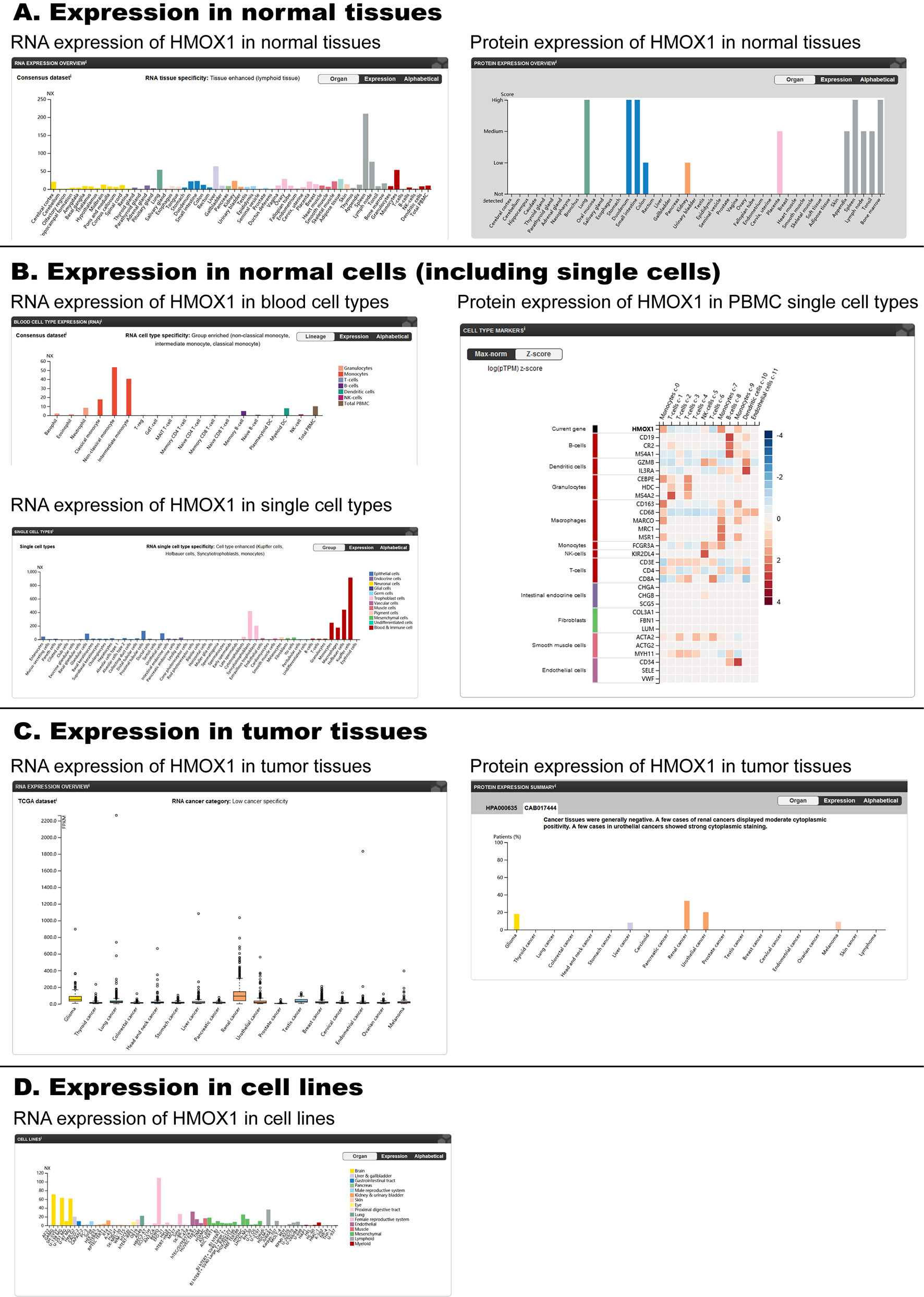

Supplement: Supplementary Figure 2 — The comprehensive illustration of HMOX1 expression in different tissues and cell types downloading from the HPA database (https://www.proteinatlas.org/). (A) The expression of HMOX1 in normal tissues. (B) The expression of HMOX1 in normal cells (including single cells). (C) The expression of HMOX1 in tumor tissues. (G) The expression of HMOX1 in cell lines. [file Image_2.JPEG]

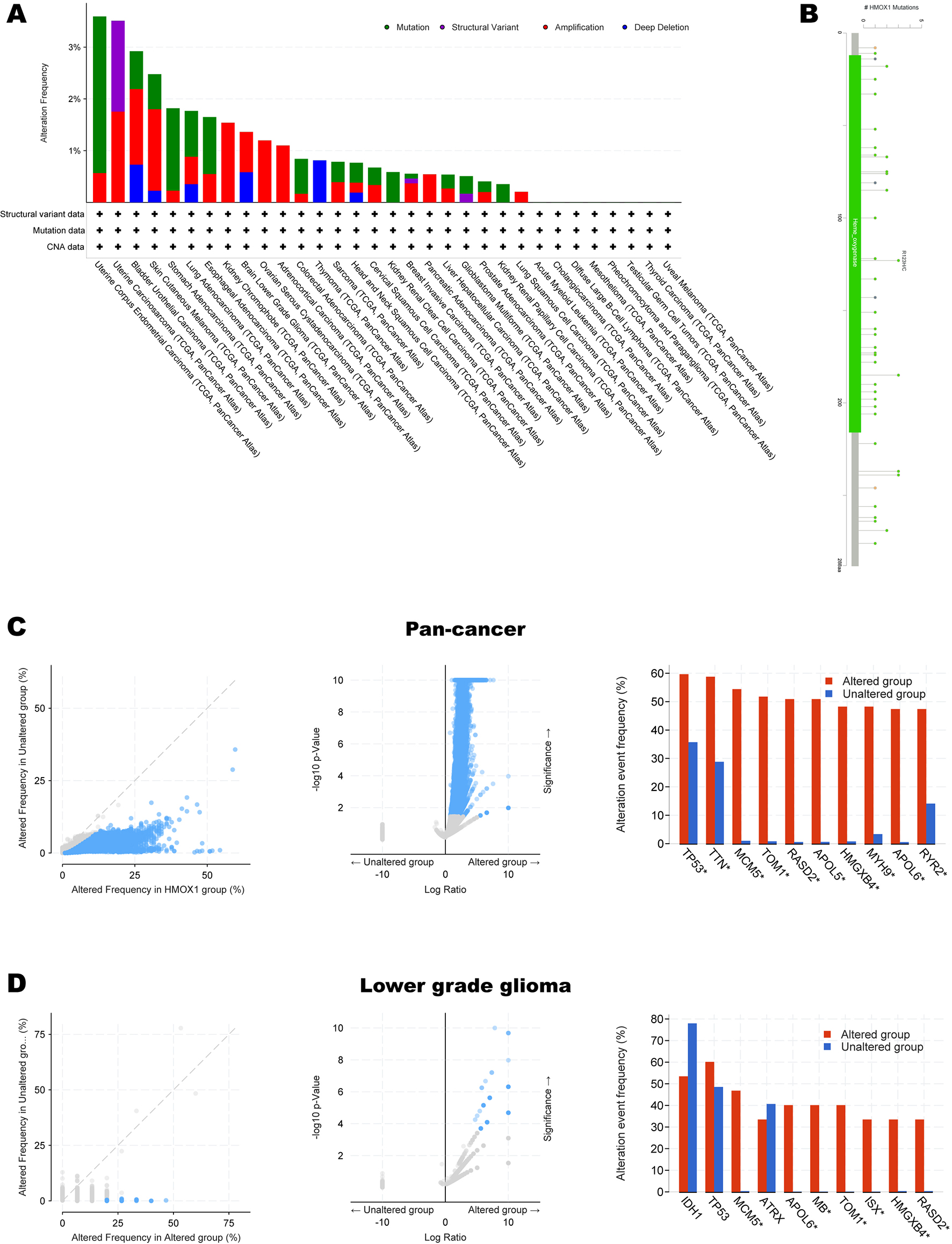

Supplement: Supplementary Figure 3 — Mutational profiles of HMOX1. (A) Summary of HMOX1 mutations in TCGA pan-cancers. (B) The general mutation count of HMOX1. (C,D) Differential altered genes between HMOX1-altered and HMOX1-unaltered groups in pan-cancer and LGG cohort. *p < 0.05. CNV, copy number variation. [file Image_3.JPEG]

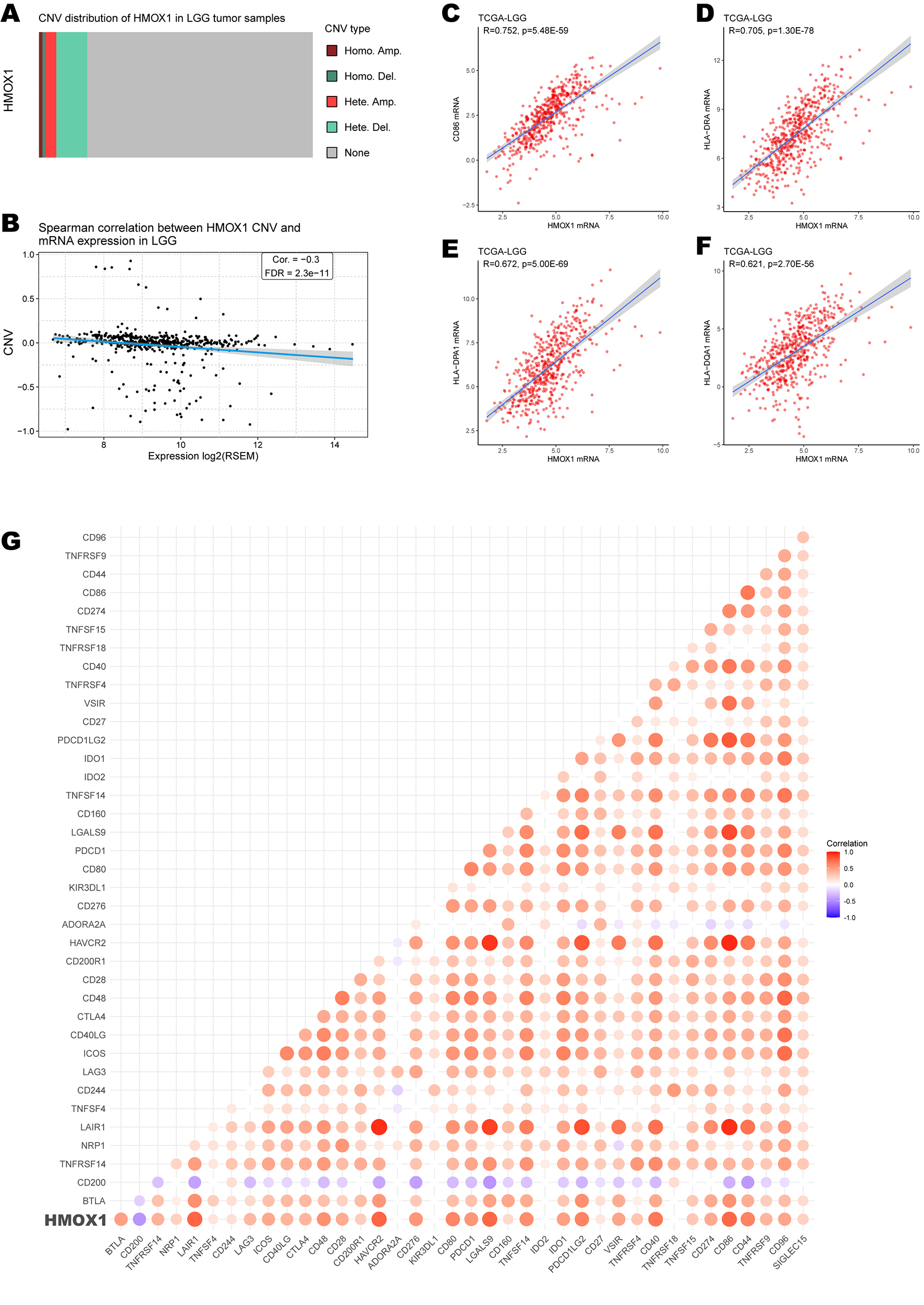

Supplement: Supplementary Figure 4 — (A) CNV distribution of HMOX1 in LGG tumor samples. (B) Spearman correlation between HMOX1 CNV and mRNA expression in LGG. (C–F) Correlations between HMOX1 and M2b macrophage markers. (G) Correlations between HMOX1 expression and recognized immune checkpoints. ∗p < 0.05, ∗∗p < 0.01, ∗∗∗p < 0.001. CNV, copy number variation; Homo. Amp., homogenous amplification; Homo. Del., homogenous deletion; Hete. Amp., heterogenous amplification; Hete. Del., heterogenous deletion. [file Image_4.JPEG]

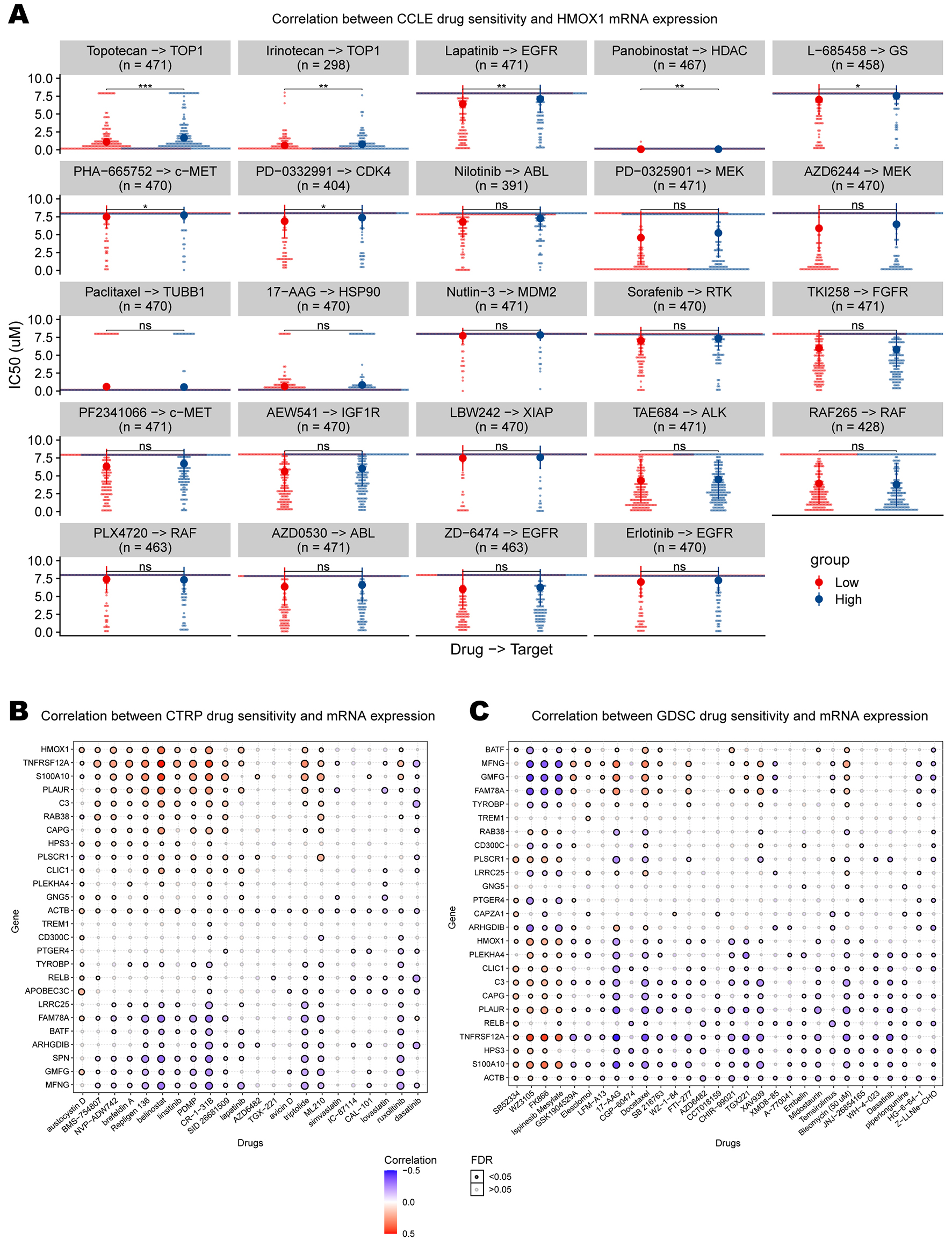

Supplement: Supplementary Figure 5 — Prediction of drug responses in the (A) Cancer Cell Line Encyclopedia (CCLE), (B) Cancer Therapeutics Response Portal (CTRP), and (C) Genomics of Drug Sensitivity in Cancer (GDSC) banks. *p < 0.05. [file Image_5.JPEG]
