# Supplementary material for: Heme Oxygenase-1 Predicts Risk Stratification and Immunotherapy Efficacy in Lower Grade Gliomas
Source: Front Cell Dev Biol. 2021 Nov 9;9:760800. doi: 10.3389/fcell.2021.760800 (PMC8631111; doi:10.3389/fcell.2021.760800)
Supplement: Supplementary file 6 [file Table_1.DOCX]

**Table S1.** Completed and ongoing clinical trials related to heme oxygenase-1*.*

| **NCT Number** | **Title** | **Status** | **Conditions** | **Phases** | **Enrollment** | **Start Date** | **Completion Date** | **Locations** |
| --- | --- | --- | --- | --- | --- | --- | --- | --- |
| NCT02232308 | Modulation of Heme Oxygenase 1 by Nizatidine and Lisinopril in Healthy Subjects | Completed | Gastroparesis | Phase 1 | 24 | Jul-14 | Sep-14 | Mayo Clinic in Rochester, Rochester, Minnesota, United States\|Mayo Clinic, Rochester, Minnesota, United States |
| NCT04070014 | Relationship Between Heme Oxygenase 1 Enzyme Level and Coronary SYNTAX Score | Completed | Coronary Artery Disease\|Oxidative Stress |  | 159 | 1-Oct-19 | 30-Mar-20 | Yeditepe University Hospital, Istanbul, Turkey |
| NCT01430156 | Induction of HO-1; a Therapeutic Approach to Reduce Ischaemia Reperfusion Injury (IRI) Following Deceased Donor Renal Transplantation | Completed | Graft Failure\|Ischemia-reperfusion Injury | Phase 3 | 40 | Jan-12 | Aug-13 | Royal Infirmary of Edinburgh/ University of Edinburgh, Edinburgh, Lothian, United Kingdom |
| NCT00895167 | The Effects of Oral Curcumin on Heme Oxygenase-1 (HO-1) in Healthy Male Subjects | Completed | Healthy | Phase 1 | 12 | Jan-09 | Aug-09 | Medical University of Vienna, Department of Clinical Pharmacology, Vienna, Austria |
| NCT03111589 | Monocytic Expression of Heme Oxidase-1 (HO-1) in Sickle Cell Patients and Correlation With the Humoral Immune Response to Vaccine and With Allo-immunization. | Completed | Sickle Cell Disease | Not Applicable | 102 | Oct-16 | Oct-18 | CHU Brugmann, Brussels, Belgium\|HUDERF, Brussels, Belgium |
| NCT02314780 | The Effects of Intravenous Heme Arginate on Heme Oxygenase-1 Expression (HO-1) and Oxidative Stress in the Human Heart | Completed | Myocardial Ischemia | Phase 2 | 31 | 29-Mar-15 | 13-Jun-17 | Medical University of Vienna, Vienna, Austria |
| NCT00882804 | Hemin in Healthy Subjects | Completed | Healthy Volunteers | Phase 1 | 10 | Feb-09 | Apr-09 | Mayo Clinic, Rochester, Minnesota, United States |
| NCT00969670 | Correlation of Particulate Matter and Heme Oxygenase-1 Protein Activity in Asthmatic Children by Induced Sputum Analysis. | Unknown status | Asthma |  | 100 | Oct-09 |  | Pulmonary Laboratory of Tel Aviv Sourasky Medical Center, Tel Aviv, Israel |
| NCT01768507 | Reresveratrol Administered to Healthy Male Subjects | Completed | Healthy Subjects\|Heme Oxygenase | Not Applicable | 10 | Mar-11 | Mar-11 | Medical University of Vienna, Vienna, Austria |
| NCT00682370 | Effects of Heme Arginate in Healthy Male Subjects | Completed | Healthy Subjects\|Heme Oxygenase\|Genetic Polymorphism | Phase 1 | 15 | Oct-07 | Jul-08 | Medical University of Vienna, Vienna, Austria |
| NCT00483587 | Does Heme Oxygenase-1 Induction Ameliorate Cardiac Injury After Myocardial Infarction? | Completed | Acute Myocardial Infarction | Phase 1\|Phase 2 | 15 | Jul-07 | Feb-10 | University Medical Centre Groningen, Groningen, Netherlands |
| NCT02142699 | Heme Arginate in Cardiac Surgery Patients | Unknown status | Acute Kidney Injury | Phase 2 | 20 | Jul-14 | Nov-14 | Royal Infirmary of Edinburgh, Edinburgh, Midlothian, United Kingdom |
| NCT02359240 | HO-1 and Muscle Mitochondrial Dysfunction in Sepsis | Unknown status | Sepsis\|Atrophy |  | 50 | Dec-14 | Aug-16 | Anesthesiology and Intensive Care, Changhai Hospital, Shanghai, Shanghai, China |
| NCT01140685 | Analysis of Anti-inflammatory and Antioxidant Pathways in Lung Diseases by Haem Oxygenase-1 (HO-1) in Induced Sputum and Carbon Monoxide (CO) in Exhaled Air | Unknown status | Lung Diseases |  | 70 | Dec-09 |  |  |
| NCT01206582 | A Pilot Study of Hemin Therapy for Gastroparesis (Diabetes Mellitus) | Completed | Gastroparesis\|Diabetes Mellitus | Phase 2 | 20 | May-10 | Dec-14 | Mayo Clinic, Rochester, Minnesota, United States |
| NCT03893799 | A Study With RBT-1, in Healthy Volunteers and Subjects With Stage 3-4 Chronic Kidney Disease | Active, not recruiting | Acute Kidney Injury | Phase 1 | 42 | 27-Aug-19 | 31-Dec-20 | Riverside Clinical Research, Edgewater, Florida, United States |
| NCT01129466 | Effects of 2 Different Broccoli Sprout Containing Supplements on Nasal Cells in Healthy Volunteers | Completed | Healthy Adult Volunteers | Not Applicable | 11 | May-10 | Aug-10 | UNC Center for Environmental Medicine, Asthma and Lung Biology, Chapel Hill, North Carolina, United States |
| NCT01329003 | DNA-damage Pathways in Workers Exposed to Silica (Caesar Stone) | Unknown status | Silicosis |  | 150 | Sep-11 | Sep-13 | Pulmonary Laboratory of Tel Aviv Sourasky Medical Center, Tel Aviv, Israel |
| NCT04411758 | Propolis for Patients With Chronic Kidney Disease. | Recruiting | Chronic Kidney Diseases\|Inflammation | Not Applicable | 60 | 12-Jan-21 | 20-Dec-21 | Denise Mafra, Niterói, Rio De Janeiro, Brazil |
| NCT04072861 | A Study With SnPP, in Healthy Volunteers and Subjects With Stage 3-4 Chronic Kidney Disease | Completed | Acute Kidney Injury | Phase 1 | 42 | 31-Mar-19 | 31-Oct-20 | Riverside Clinical Research, Edgewater, Florida, United States |
| NCT02191813 | Study to Determine the Ability of Seresis to Act as a Skin Protection Agent in Healthy Young Female Volunteers | Completed | Healthy | Phase 2 | 48 | Mar-00 |  |  |
| NCT04458116 | Effects of Curcumin on Markers of Cardiovascular Risk in Patients With CAD | Not yet recruiting | Coronary Artery Disease\|Oxidative Stress\|Inflammation | Not Applicable | 30 | 10-Mar-22 | 20-Nov-22 | Denise Mafra, Rio de Janeiro, RJ, Brazil |
| NCT00856817 | Heme Oxygenase (HO) Activity and Adenosine Induced Vasodilation | Terminated | Atherosclerotic Cardiovascular Disease | Phase 4 | 6 | Mar-09 |  | Radboud University Nijmegen Medical Centre, Nijmegen, Gelderland, Netherlands |
| NCT01038050 | Study Effects of Ginkgo Biloba Extract on Endothelial Cell Function and Genetic Effects on the Response to Ginkgo Biloba Extract in Diabetic Patients With Stable Coronary Artery Disease | Unknown status | Type 2 Diabetes Mellitus\|Coronary Artery Disease | Phase 4 | 60 | Oct-09 | Dec-10 | Taipei Veterans General Hospital, Taipei, Taiwan |
| NCT00452764 | Regulatory T Cells in COPD | Completed | Chronic Obstructive Pulmonary Disease |  | 50 | Jan-07 | Nov-07 | University Medical Center Groningen, Groningen, Netherlands |
| NCT04195438 | Postoperative Heme Oxygenase Induction and Carbon Monoxide Production as a Novel Method to Assess Hepatic Regeneration and Predict Hepatic Related Morbidity After Partial Hepatectomy | Recruiting | Liver Cancer | Not Applicable | 30 | 7-May-14 | 31-Dec-23 | University of Maryland Baltimore Washington Medical Center, Glen Burnie, Maryland, United States |
| NCT00842205 | Role of Heme Oxygenase in the Pathogenesis of Hepatocellular Injury in Chronic Hepatitis C Virus (HCV) Infection | Unknown status | Chronic HCV Infection\|Nonalcoholic Steatohepatitis | Not Applicable | 150 | Jan-07 | Dec-09 | Cetral Military Hospital, Prague, Czech Republic |
| NCT03033810 | FFR Versus iFR in Assessment of Hemodynamic Lesion Significance | Unknown status | Hemodynamics\|Coronary Circulation\|Tomography, Optical Coherence\|Endothelial Cells\|Polymorphism, Genetic |  | 200 | Jan-17 | Dec-19 | II. interni klinika VFN, Praha, Czech Republic |
| NCT01942291 | Short-term Effect of Extended-release Niacin on Endothelial Function. | Completed | Hypoalphalipoproteinemia | Phase 4 | 18 | Mar-12 | Jun-13 | University of Campinas, Campinas, SP, Brazil |
| NCT01461512 | Heme Arginate in Functional Magnetic Resonance Imaging (fMRI) | Unknown status | Ischemia-reperfusion Injury | Phase 2 | 16 | Jan-09 | Dec-11 | Medical University of Vienna, Department of Clinical Pharmacology, Vienna, Austria |
| NCT03648970 | Indonesia Pravastatin to Prevent Preeclampsia Study | Unknown status | Pre-Eclampsia | Phase 2 | 280 | 1-Mar-18 | 1-Dec-20 | Sanglah General Hospital, Denpasar, Bali, Indonesia\|Dr. Moewardi Hospital, Surakarta, Central Java, Indonesia\|Ramelan Naval Hospital, Surabaya, East Java, Indonesia\|Dr. Soetomo Hospital, Surabaya, East Java, Indonesia\|Adam Malik General Hospital, Medan, North Sumatra, Indonesia\|Dr. Wahidin Sudirohusodo General Hospital, Makasar, South Sulawesi, Indonesia\|Hasan Sadikin General Hospital, Bandung, West Java, Indonesia |
| NCT04900532 | Effects of Supplementation With Tocotrienol on Chronic Kidney Disease Patients | Active, not recruiting | Chronic Kidney Diseases\|Hemodialysis\|Microbiota\|Oxidative Stress\|Inflammation | Not Applicable | 61 | 1-May-19 | 31-Mar-22 | Denise Mafra, Rio de Janeiro, RJ, Brazil |
| NCT04552821 | Study of Biomarkers in Patients of Sepsis Complicated With Acute Respiratory Distress Syndrome (ARDS) | Recruiting | Sepsis\|Acute Respiratory Distress Syndrome |  | 90 | 2-Jun-20 | 30-Jun-22 | Tianjin NanKai hospital, Tianjin, Tianjin, China |
| NCT04564833 | Effect of RBT-1 on Preconditioning Response Biomarkers in Subjects Undergoing CABG and/or Cardiac Valve Surgery | Recruiting | AKI | Phase 2 | 126 | 30-Apr-21 | 30-Nov-22 | MedStar Health Research Institute, Inc., Hyattsville, Maryland, United States\|University of Texas Southwestern Medical Center, Dallas, Texas, United States\|Monash Medical Centre, Clayton, Victoria, Australia\|Flinders Medical Centre, Bedford Park, Australia\|The Alfred Hospital, Melbourne, Australia |
| NCT04873479 | Effect of S-ketamine Anesthetic on Inflammatory Response in Septic Patients Undergoing Abdominal Surgery | Not yet recruiting | Sepsis\|Acute Lung Injury | Not Applicable | 50 | 15-May-21 | 30-Oct-23 | Tianjin Nankai Hospital, Tianjin, Tianjin, China |
| NCT03630029 | RBT-1 Phase 1b Clinical Trial in Healthy Volunteers and Subjects With CKD | Withdrawn | Acute Kidney Injury | Phase 1 | 0 | 15-Sep-18 | 31-Mar-19 |  |
| NCT04848792 | Treatment Strategy to Enhance Nrf2 Signaling in Older Adults | Not yet recruiting | Aging Problems | Not Applicable | 30 | 1-Jul-21 | 30-Jun-23 | Northern Arizona University, Flagstaff, Arizona, United States |
| NCT00978094 | Validation of a Novel Sham Cervical Spinal Manipulation Procedure | Completed | Chronic Neck Pain |  | 80 | Feb-09 | Oct-10 | Canadian Memorial Chiropractic College, Toronto, Ontario, Canada |
| NCT01773512 | The Prediction of Extent and Risk Profile of Coronary Atherosclerosis and Their Changes During Lipid-lowering Therapy Based on Non-invasive Techniques | Unknown status | Coronary Artery Disease | Phase 4 | 60 | Jun-12 | Jun-15 | Loyola University Hospital, Maywood, Illinois, United States\|The University of Iowa, Iowa City, Iowa, United States\|General University Hospital, Prague, Czech Republic |
| NCT03309631 | Clinical Validation of ThyroidPrint: A Gene Expression Signature for Diagnosis of Indeterminate Thyroid Nodules | Unknown status | Indeterminate Thyroid Cytology |  | 1500 | 1-Mar-16 | 30-Jun-18 | Avantt Research, Guntersville, Alabama, United States\|Stanford University, Palo Alto, California, United States\|Lee Moffit Cancer Center, Tampa, Florida, United States\|Tulane University, New Orleans, Louisiana, United States\|Universidad de Cincinnati, Cincinnati, Ohio, United States\|University of Texas MD Anderson Cancer Center, Houston, Texas, United States |
| NCT03061318 | Validation of a Multi-Genetic Test for the Diagnosis of Indeterminate Thyroid Nodules | Completed | Indeterminate Thyroid Cytology |  | 3100 | 8-Aug-15 | 30-Aug-17 | Centro de Diagnóstico Plaza Italia, Santiago, Region Metropolitana, Chile\|Clínica Alemana de Santiago, Santiago, Region Metropolitana, Chile\|Hospital Clínico de la Pontificia Universidad Católica de Chile, Santiago, Region Metropolitana, Chile\|Clínica San Carlos de Apoquindo, Santiago, Región Metropolitana, Chile\|Clínica Santa María, Santiago, Región Metropolitana, Chile\|Fundación Arturo López Pérez, Santiago, Región Metropolitana, Chile\|Hospital del Salvador, Santiago, Región Metropolitana, Chile\|Hospital San Juan de Dios, Santiago, Región Metropolitana, Chile\|Hospital Clínico de la Universidad de Chile, Santiago, Chile |
| NCT04647201 | Study of Biomarkers in Patients of Sepsis Complicated With Gastrointestinal Dysfunction | Not yet recruiting | Sepsis\|Gastrointestinal Dysfunction |  | 90 | 23-Nov-20 | 30-Jun-22 | Tianjin Nankai Hospital, Tianjin, China |
| NCT01338064 | Oxidative Stress Biomarkers to Monitor and Early Detect Health Impairment in Workers Exposed to Silica (Caesar Stone) | Unknown status | Silicosis |  | 150 | Sep-11 | Sep-13 | Pulmonary Laboratory of Tel Aviv Sourasky Medical Center, Tel Aviv, Israel |
| NCT02147522 | A Helping Hand Among Low-Income Patients | Completed | Depression\|Diabetes\|Heart Disease | Not Applicable | 348 | Oct-13 | Sep-16 | El Monte Comprehensive Health Center, El Monte, California, United States\|H. Claude Hudson Comprehensive Health Center, Los Angeles, California, United States\|Edward R. Roybal Comprehensive Health Center, Los Angeles, California, United States |
| NCT03195205 | Identification to Elimination in HCV-Infected Individuals | Completed | Hepatitis C | Early Phase 1 | 3051 | 1-Jun-17 | 20-Apr-20 | TruCare Internal Medicine & Infectious Diseases, DuBois, Pennsylvania, United States |
| NCT00272311 | Aspirin Dose and Atherosclerosis in Patients With Metabolic Syndrome | Completed | Cardiovascular Diseases\|Metabolic Syndrome X\|Atherosclerosis | Phase 4 | 70 | Oct-06 | Jan-09 | HeartDrug Research, LLC, Towson, Maryland, United States |
| NCT01855841 | Hemin to Prevent Post-ERCP (Endoscopic Retrograde Cholangiopancreatography) Acute Pancreatitis | Completed | Post-ERCP Acute Pancreatitis | Phase 2 | 284 | Apr-12 | Jun-21 | CHU Brugmann, Brussels, Belgium\|Erasme Hospital, Université Libre de Bruxelles (ULB), Brussels, Belgium\|Centre Hospitalier de Jolimont-Lobbes, Haine-St-Paul, Belgium\|Hôpital Ambroise Paré, Mons, Belgium\|ISPPC CHU Vésale, Montigny Le tilleul, Belgium\|National Taiwan University Hospital, Taipei, Taiwan |
| NCT03206164 | HealthMatters@24/7 eLearning for People Supporting Adults With Intellectual and Developmental Disabilities | Active, not recruiting | Obesity\|Hypertension\|Hypercholesterolemia\|Hyperglycemia | Not Applicable | 60 | 1-May-20 | 31-Dec-21 | UIC, Chicago, Illinois, United States |
| NCT01612234 | Saturated Fat Versus Monounsaturated Fat and Insulin Action | Completed | Obesity | Not Applicable | 70 | Apr-10 | Nov-15 | The Unversity of Vermont Clinical Research Center at Fletcher Allen Health Care, Burlington, Vermont, United States |
| NCT04936672 | Effects of Brisk Walking Combined With Tai Chi Chuan on Health-Related Physical Fitness and Selected Health Parameters Among Older Chinese Women | Not yet recruiting | Walking | Not Applicable | 104 | 10-Jul-21 | 30-Oct-21 | Puyang Aged Institution, Puyang, Henan, China |
| NCT04600258 | Chocolate for Patients With Chronic Kidney Disease | Recruiting | Chronic Kidney Disease | Not Applicable | 48 | 1-Oct-20 | 1-Dec-22 | Denise Mafra, Rio de Janeiro, RJ, Brazil\|Denise Mafra, Rio de Janeiro, RJ, Brazil |
| NCT04413266 | Effects of Curcumin Supplementation in Patients With Chronic Kidney Disease on Peritoneal Dialysis | Recruiting | Chronic Kidney Diseases\|Peritoneal Dialysis\|Hemodialysis | Not Applicable | 30 | 10-Oct-20 | Oct-21 | Denise Mafra, Rio de Janeiro, RJ, Brazil |
| NCT03475017 | Effects of Curcumin in Patients in Chronic Kidney Disease | Active, not recruiting | Chronic Kidney Diseases | Not Applicable | 30 | 22-Feb-18 | 30-Dec-21 | Denise Mafra, Rio de Janeiro, RJ, Brazil |
| NCT02205775 | Rosuvastatin for Reduction of Myocardial Damage and Systemic Inflammation During Coronary Angioplasty | Terminated | Stable Coronary Artery Disease Undergoing PCI | Phase 3 | 280 | May-10 | Jun-12 | SS. Annunziata Hospital, Chieti, CH, Italy\|Fondazione IRCCS Policlinico S. Matteo, Pavia, PV, Italy\|A.O. S. Anna e S. Sebastiano - II Università di Napoli, Caserta, Italy\|Azienda ASL 6 - P. Ospedaliero Livorno, Livorno, Italy\|Ospedale Civile G. Fornaroli, Magenta, Italy\|Azienda Ospedaliera - Ospedale San Paolo, Milano, Italy |
| NCT02433925 | Resveratrol's Effects on Inflammation and Oxidative Stress in Chronic Kidney Disease | Completed | Chronic Renal Insufficiency | Phase 3 | 20 | Jan-13 | Dec-14 |  |
| NCT01232205 | Antioxidant Supplementation in Pregnant Women | Completed | Pregnant Women\|Preeclampsia | Phase 2\|Phase 3 | 168 | Jun-01 | Mar-10 | Cipto Mangunkusumo National Hospital, Jakarta, Indonesia |
| NCT02213523 | ABG Oxidative Stress Study Protocol-1 | Completed | Healthy | Not Applicable | 60 | Sep-12 | Dec-13 | Quality of Life, Buena Park, California, United States\|Southbay Pharma Research, Buena Park, California, United States |
| NCT04205123 | Biological, Genetic and Environmental Involved in the Complications of Sickle Cell Disease | Recruiting | Sickle Cell Disease |  | 200 | 20-Oct-14 | 1-Jan-25 | Erasme Hospital, Brussels, Belgium |
| NCT04344769 | Characterization of the Nrf2 Response in Patients With Autosomal Dominant Polycystic Kidney Disease (ADPKD) | Recruiting | Autosomal Dominant Polycystic Kidney Disease |  | 40 | 4-Oct-19 | 31-Mar-22 | Mayo Clinic in Rochester, Rochester, Minnesota, United States |
| NCT00691613 | Infusion of a Single Dose of Erythropoietin to Prevent Injury in an Ischemia Reperfusion Forearm Model | Unknown status | Ischemia-Reperfusion Injury\|Myocardial Infarction | Not Applicable | 12 | Jul-10 | Dec-11 | Clinical Research Centre Nijmegen, Nijmegen, Gelderland, Netherlands |
| NCT01335971 | Broccoli Sprout Extracts Trial to See if NRF2 is Enhanced by Sulforaphane Treatment in Patients With COPD | Completed | COPD | Phase 2 | 89 | Sep-10 | Jun-15 | Johns Hopkins School of Medicine, Baltimore, Maryland, United States\|University at Baffalo, The State University of New York, Buffalo, New York, United States\|Temple University, Philadelphia, Pennsylvania, United States |
| NCT02819856 | SPI-1005 for Prevention and Treatment of Tobramycin Induced Ototoxicity | Enrolling by invitation | Ototoxicity | Phase 2 | 80 | 21-Jul-17 | Dec-21 | Medical University of South Carolina, Charleston, South Carolina, United States |
| NCT03685552 | Safety Evaluation of a Diet and Nutritional Supplementation Program- Purify 2.0 | Completed | Gastrointestinal Symptoms | Not Applicable | 38 | 3-Aug-17 | 13-Sep-17 | The Hughes Center for Research and Innovation, Lehi, Utah, United States |
| NCT02739438 | The Study of ELEctronic Cigarette Toxicity in a Human Model in Vivo Model of Inflammation and Vascular Dysfunction | Unknown status | Acute Lung Injury |  | 30 | 1-Feb-17 | Dec-19 | Queens University, Belfast, N Ireland, United Kingdom |
| NCT03262363 | Curcumin on NFE2L2 Gene Expression, Antioxidant Capacity and Renal Function According to rs35652124 in Diabetic Nephropathy | Unknown status | Chronic Kidney Diseases\|Diabetes Mellitus, Type 2\|Polymorphism | Phase 2\|Phase 3 | 176 | 1-Aug-18 | 30-Apr-19 | Umae Hospital de Especialidades, Guadalajara, Jalisco, Mexico |
| NCT02876653 | Accelerated Aging in Middle-Aged Men With Sleep-disordered Breathing. | Completed | Obstructive Sleep Apnea | Not Applicable | 53 | Mar-11 | Jan-14 | Henri Mondor Hospital, Creteil, France |
| NCT01625130 | Effect of Sulforaphane-rich Broccoli Sprout Homogenate on Ozone Induced Inflammation Through Modulation of NRF2 | Completed | Healthy | Not Applicable | 16 | Oct-13 | Jun-15 | UNC Center for Environmental Medicine, Asthma and Lung Biology, Chapel Hill, North Carolina, United States |
| NCT02264496 | Prospective Randomised Trial of Exercise and / or Antioxidants in COlorectal Cancer Patients Undergoing Surgery. | Completed | Colorectal Cancer | Not Applicable | 24 | Mar-14 | Nov-15 | Academic Surgical Unit, Castle Hill Hospital, Hull, East Yorkshire, United Kingdom |
| NCT03049046 | CC100: Phase 1 Multiple-Dose Safety and Tolerability in Subjects With ALS | Unknown status | Amyotrophic Lateral Sclerosis | Phase 1 | 21 | 7-Apr-17 | 30-Mar-18 | Indiana University, IU Health Physicians Neurology, Indianapolis, Indiana, United States |
| NCT03926819 | A Study to Assess the Safety and Pharmacokinetics of HBI-002, an Oral Carbon Monoxide Therapeutic, in Healthy Volunteers | Not yet recruiting | Anemia, Sickle Cell | Phase 1 | 20 | May-21 | Oct-21 |  |
| NCT02561481 | Sulforaphane Treatment of Children With Autism Spectrum Disorder (ASD) | Completed | Autism Spectrum Disorder | Phase 1\|Phase 2 | 60 | Dec-15 | Jan-20 | University of Massachusetts Medical School, Worcester, Massachusetts, United States |
| NCT02752789 | Impact of Allo- and Autoantibodies on Chronic Cardiac Allograft Function | Completed | Pediatric Heart Transplantation\|Pediatric Heart Transplant Recipients |  | 407 | 15-Jul-14 | 1-Nov-19 | Emory University School of Medicine, Atlanta, Georgia, United States\|Children's Hospital Boston, Boston, Massachusetts, United States\|St. Louis Children's Hospital, Saint Louis, Missouri, United States\|Columbia University Medical Center, New York, New York, United States\|Children's Hospital at Montefiore, New York, New York, United States\|Children's Hospital of Philadelphia, Philadelphia, Pennsylvania, United States\|Children's Hospital of Pittsburgh, Pittsburgh, Pennsylvania, United States\|Monroe Carell Jr. Children's Hospital, Nashville, Tennessee, United States\|Hospital for Sick Children, Toronto, Canada |
| NCT01732718 | Effect of Atorvastatin on Endothelial Dysfunction and Albuminuria in Sickle Cell Disease | Completed | Sickle Cell Disease\|Sickle Cell Nephropathy | Phase 2 | 13 | Sep-13 | 9-Jan-18 | UNC School of Medicine Clinical&Translational Research Ctr, Chapel Hill, North Carolina, United States |
| NCT04011072 | Far Infrared Therapy on Arteriovenous Fistulas in Hemodialysis Patients | Recruiting | Arterio-venous Fistula | Not Applicable | 187 | 3-Oct-19 | Aug-24 | Frederiksberg Hospital, Frederiksberg, Denmark\|Herlev Hospital, Herlev, Denmark\|Hilleroed Hospital, Hillerød, Denmark\|Holbæk Hospital, Holbæk, Denmark\|Hvidovre Hospital, Hvidovre, Denmark\|Rigshospitalet, København, Denmark\|Nykøbing Falster Hospital, Nykøbing Falster, Denmark\|Roskilde Hospital, Roskilde, Denmark\|Slagelse Hospital, Slagelse, Denmark |
| NCT03029351 | GLP-1 Receptor Agonist Therapy and Albuminuria in Patients With Type 2 Diabetes | Unknown status | Type2 Diabetes\|Kidney Diseases | Phase 4 | 90 | Feb-17 | Feb-20 |  |
| NCT01257191 | A Study to Compare the Effects of Different Sized Particles on Cells in the Nose | Completed | Hypersensitivity, Immediate\|Atopic Hypersensitivity | Phase 1 | 25 | Apr-10 | Jan-12 | University of California, Los Angeles, Los Angeles, California, United States |
| NCT02138045 | Treatment of Diabetic Neuropathy With Liraglutide | Unknown status | Diabetes Mellitus, Type 1\|Type 1 Diabetes Mellitus | Not Applicable | 40 | May-14 | Feb-17 | Mech-Sense, Department of Medical Gastroenterology, Aalborg University Hospital, Aalborg, Jutland, Denmark |
| NCT02683863 | Pharmacokinetics of DMF and the Effects of DMF on Exploratory Biomarkers | Completed | Multiple Sclerosis | Phase 4 | 20 | Aug-15 | 31-Jan-17 | Multiple Sclerosis Center of Northeastern New York, Latham, New York, United States |
| NCT04805333 | Phase 1 Dose Escalation of ArtemiCoffee | Recruiting | Ovarian Cancer | Phase 1 | 18 | 26-Mar-21 | 31-Dec-22 | University of Kentucky, Lexington, Kentucky, United States |
| NCT04937855 | The Mechanism of lncRNA NEAT1 in Alleviating Acute Respiratory Distress Syndrome Through miR-27b Regulated Nrf2 Pathway | Enrolling by invitation | Acute Respiratory Distress Syndrome\|Inflammation |  | 425 | 1-Jul-21 | 31-Dec-23 | Department of Respiratory and Critical Care Medicine, Beijing Anzhen Hospital, Capital Medical University, Beijing Institute of Heart, Lung and Blood Vessel Diseases, Beijing, Beijing, China |
| NCT03494764 | Hyperbaric Oxygen Therapy for Ulcerative Colitis Flares | Completed | Colitis, Ulcerative | Phase 2 | 39 | 7-Sep-17 | 31-Mar-20 | UC San Diego Health Systems, La Jolla, California, United States\|University of California San Diego, San Diego, California, United States\|University of Maryland, Baltimore, Maryland, United States\|Mayo Clinic, Rochester, Minnesota, United States\|Dartmouth-Hitchcock Medical Center, Lebanon, New Hampshire, United States\|NYU Langone Medical Center, New York, New York, United States\|University of Texas Southwestern Medical Center, Dallas, Texas, United States\|Virginia Mason Memorial Hospital, Yakima, Washington, United States |
| NCT02346071 | Acceptance and Commitment Group Therapy for Adolescents With a Range of Functional Somatic Syndromes | Completed | Somatization Disorder\|Somatoform Disorders | Not Applicable | 91 | 30-Jan-15 | 21-Nov-19 | Research Clinic for Functional Disorders and Psychosomatics, Aarhus, Denmark |
| NCT00740714 | Effects of Coenzyme Q10 (CoQ) in Parkinson Disease | Terminated | Parkinson Disease | Phase 3 | 600 | Dec-08 | Aug-11 | University of Alabama, Birmingham, 350 Sparks Center, 1720 7Th Avenue South, Birmingham, Alabama, United States\|Barrow Neurological Clinics At St Joseph'S Hospital & Medical Center, 500 West Thomas Road Suite 720, Phoenix, Arizona, United States\|Mayo Clinic Arizona, 13400 East Shea Boulevard, Desk 34 3B, Scottsdale, Arizona, United States\|Sunhealth Research Institute, 10515 West Santa Fe Drive, Sun City, Arizona, United States\|The Parkinson'S & Movement Disorder Institute, 9940 Talbert Avenue, Suite 204, Fountain Valley, California, United States\|University of California Irvine, 100 Irvine Hall, Irvine, California, United States\|University of California San Diego, Alzheimer'S Disease Research Center, 9500 Gilman Drive, La Jolla, California, United States\|UCLA Medical Center, 710 Westwood Plaza, A-253, Los Angeles, California, United States\|UC Davis Dept of Neurology, 4860 Y Street, Suite 3700, Sacramento, California, United States\|The Parkinson's Institute, 675 ALMANOR AVENUE, Sunnyvale, California, United States\|Department of Neurology/Mail Stop B185, 12631 East 17Th Avenue Room 5209, Academic Office 1 Po Box 6511, Aurora, Colorado, United States\|Colorado Neurological Institute, 701 East Hampden Avenue, Suite 510, Littleton, Colorado, United States\|The Institute For Neurodegenerative Disorders, 60 Temple Street, Suite 8B, New Haven, Connecticut, United States\|University of Florida, McKnight Brain Institute Po Box 100236, 100 S Newell Drive L3-100, Gainsville, Florida, United States\|University of Miami, 1501 North West 9Th Avenue Second Floor, Department of Neurology D4-5, Miami, Florida, United States\|University of South Florida, 4 Columbia Drive, Suite 410, Tampa, Florida, United States\|Emory University School of Medicine, Wesley Woods Health Center, 1841 Clifton Road NE Room 328, Atlanta, Georgia, United States\|Movement Disorders Program, Department of Neurology, Medical College of Georgia, Augusta, Georgia, United States\|Northwestern University, 710 North Lake Shore Drive, Chicago, Illinois, United States\|Rush University Medical Center Department of Neurological Sciences, 1725 West Harrison Suite 755, Chicago, Illinois, United States\|University of Chicago, 5841 South Maryland Avenue, Mc2030, Chicago, Illinois, United States\|Indiana University School of Medicine, Outpatient Clinical Research Facility, 535 Barnhill Drive Room #150, Indianapolis, Indiana, United States\|University of Iowa Hospitals, 2133 Rcp Department of Neurology, 200 Hawkins Drive, Iowa City, Iowa, United States\|The University of Kansas Medical Center, Department of Neurology Ms #2012, 3599 Rainbow Boulevard, Kansas City, Kansas, United States\|University of Louisville, Movement Disorder Clinic, Frazier Rehab, 220 Abraham Flexner, Suite 606, Louisville, Kentucky, United States\|Ochsner Clinic Foundation, 1514 Jefferson Highway, Dept of Neurology 7Th Floor, New Orleans, Louisiana, United States\|Lsuhsc Shreveport, Department of Neurology, 1501 Kings Highway Room 3-436, Shreveport, Louisiana, United States\|University of Maryland School of Medicine, 22 South Greene Street, N4 W49-B, Baltimore, Maryland, United States\|Johns Hopkins, 601 North Caroline Street, Suite 5064, Baltimore, Maryland, United States\|Parkinson & Movement Dis Center If Maryland, 8180 Lark Brown Road, Suite 101, Elkridge, Maryland, United States\|Boston University Medical Center, Department of Neurology, 715 Albany Street C329, Boston, Massachusetts, United States\|Beth Israel Deaconess Medical Center, 330 Brookline Avenue, Shapiro 809D, Boston, Massachusetts, United States\|University of Minnesota, 420 Delaware Street SE, Mmc 295, Minneapolis, Minnesota, United States\|Washington University School of Medicine, 660 South Euclid, Box 8111, St Louis, Missouri, United States\|Albany Medical College, Parkinson'S Disease & Movement Disorders Ctr, 47 New Scottland Avenue, Albany, New York, United States\|JACOBI MEDICAL CENTER, 1400 Pelham Pkwy S, Bronx, New York, United States\|Suny Downstate Medical Center , 450 Clarkson Avenue , Box 1213, Brooklyn, New York, United States\|Northshore-Lij Health System, the Feinstein Institute Fpr Medical Research, 350 Community Drive Room 100, Manhasset, New York, United States\|Beth Israel Medical Center, 10 Union Square East, Suite 5Hh2, New York, New York, United States\|Beth Israel Medical Center, Phillips Ambulatory Care Center, 10 Union Square East Room 5Ho1, New York, New York, United States\|Parkinson'S Dis & Movement Disorders Inst, 428 East 72Nd Street, Suite 400, New York, New York, United States\|Weill Medical College of Cornell, New York, New York, United States\|Columbia University, 710 West 168Th Street, 3Rd Floor, New York, New York, United States\|University of Rochester Department of Neurology, 919 Westfall Road Building C Suite 220, Rochester, New York, United States\|Duke University Medical Center, Duke Health Center At Morreene Road, 932 Morreene Road Room 213, Durham, North Carolina, United States\|University Neurology Inc., 222 Piedmont Avenue, Suite 3200, Cincinnati, Ohio, United States\|The Cleveland Clinic Foundation, 9500 Euclid Avenue S-31, Cleveland, Ohio, United States\|Ohio State University Medical Center, 1581 Dodd Drive, 371 McCampbell Hall, Columbus, Ohio, United States\|University of Toledo , 3000 Arlington Avenue , Mail Stop 1195, Toledo, Ohio, United States\|Oregon Health & Science University, Dept of Neurology, 3181 SW Sam Jackson Park Road Op-32, Portland, Oregon, United States\|Penn State Milton S Hershey Med Center, Department of Neurology Mc H109 Room 2846, 500 University Drive Po Box 850, Hershey, Pennsylvania, United States\|University of Pennsylvania, Pennsylvania Hospital Department of Neurology, 330 South 9Th Street, Philadelphia, Pennsylvania, United States\|Neurohealth Parkinson'S Disease, Movement Disorder Center, 227 Centerville Road, Warwick, Rhode Island, United States\|Medical University of South Carolina, Charleston Memorial Hospital, 326 Calhoun Street Suite 308, Charleston, South Carolina, United States\|Semmes Murphey Clinic, 1211 Union Avenue, Suite 200, Memphis, Tennessee, United States\|Baylor College of Medicine - Parkinson'S, Disease Center and Movement Disorders Clinic, 65501 Fannin St, Suite 1801, Houston, Texas, United States\|University of Vermont , Department of Neurology Given Building C-219 , 89 Beaumont Avenue, Burlington, Vermont, United States\|Booth Gardner Parkinson'S Care Center, 13030 121St Way North East Suite 203, Kirkland, Washington, United States\|Medical College of Wisconsin, Department of Neurology, 9200 West Wisconsin Avenue, Milwaukee, Wisconsin, United States\|Un of Calgary Movement Disorders Program, Dept of Clin Neurosciences Area 3 Neurology, 3350 Hospital Dr NW Health Sciences Centre, Calgary, Alberta, Canada\|University of Alberta Glenrose Rehab Hosp, Rm 0601 Glen East, 10230 - 111 Avenue, Edmonton, Alberta, Canada\|London Health Sciences Centre, University Campus Room 10N29, 339 Windermere Road, London, Ontario, Canada\|The Ottawa Hospital-Civic Campus, 1053 Carling Avenue C2 Room 2210, Ottawa, Ontario, Canada\|Toronto Western Hospital, Univ Health Network, 399 Bathurst Street Mc 7-402, Movement Disorders Centre, Toronto, Ontario, Canada\|CHUM-HOPITAL NOTRE DAME, 1560 rue SHERBROOKE est ROOM GR 1185, PAVILLON DECHAMPS etage rez-de-chaussee, Montreal, Quebec, Canada\|University of Sherbrooke, 3001 12E Avenue Nord, Sherbrooke, Quebec, Canada\|Royal University Hospital, 103 Hospital Drive, Room 1663, Saskatoon, Saskatchewan, Canada\|Quebec Memory and Motor Skills Dis Clinic, Price Building 3Rd Floor, 65 Sainte-Anne Street, Quebec, Canada |
| NCT04114188 | Tacrolimus After rATG and Infliximab Induction Immunosuppression (RIMINI) | Completed | Renal Transplant Rejection | Phase 2 | 68 | 15-Dec-16 | 31-Dec-20 | Charité University Medicine Berlin, Berlin, Germany |
| NCT03167788 | Red Cell Rejuvenation for the Attenuation of Transfusion Associated Organ Injury in Cardiac Surgery | Withdrawn | Organ Failure, Multiple\|Inflammation\|Sepsis | Phase 2 | 0 | Dec-20 | Dec-20 | Department of Cardiovascular Sciences, Leicester, Leicestershire, United Kingdom |
