# Supplementary material for: Heme Oxygenase-1 Predicts Risk Stratification and Immunotherapy Efficacy in Lower Grade Gliomas
Source: Front Cell Dev Biol. 2021 Nov 9;9:760800. doi: 10.3389/fcell.2021.760800 (PMC8631111; doi:10.3389/fcell.2021.760800)
Supplement: Supplementary file 7 [file Table_2.DOCX]

**Table S2.** Information of the IHC results.

| ID | Target | Positive Area (%) | Mean Density | Area Density | H-Score | Age | Gender | Grade |
| --- | --- | --- | --- | --- | --- | --- | --- | --- |
| GT504-1 | HMOX1 | 38.019 | 0.088 | 0.033606 | 96.315 | 49 | Male | Ⅱ |
| GTR644 | HMOX1 | 72.439 | 0.099 | 0.071658 | 101.889 | 42 | Male | Ⅱ |
| GT647 | HMOX1 | 50.106 | 0.094 | 0.047244 | 108.464 | 44 | Male | Ⅱ |
| GT589 | HMOX1 | 58.873 | 0.12 | 0.070573 | 110.981 | 50 | Male | Ⅲ |
| GT650 | HMOX1 | 47.309 | 0.062 | 0.029265 | 120.513 | 67 | Male | Ⅲ |
