# Supplementary material for: Heme Oxygenase-1 Predicts Risk Stratification and Immunotherapy Efficacy in Lower Grade Gliomas
Source: Front Cell Dev Biol. 2021 Nov 9;9:760800. doi: 10.3389/fcell.2021.760800 (PMC8631111; doi:10.3389/fcell.2021.760800)
Supplement: Supplementary file 8 [file Table_3.DOCX]

**Table S3.** Detailed description of model genes.

| **HRG** | **LASSO coefficient** | **Description** |
| --- | --- | --- |
| *ACTB* | -0.23826973 | Actin Beta |
| *APOBEC3C* | 0.31194535 | Apolipoprotein B MRNA Editing Enzyme Catalytic Subunit 3C |
| *ARHGDIB* | -0.25451366 | Rho GDP Dissociation Inhibitor Beta |
| *BATF* | -0.04383807 | Basic Leucine Zipper ATF-Like Transcription Factor |
| *C3* | -0.01140817 | Complement C3 |
| *CAPG* | 0.18311427 | Capping Actin Protein, Gelsolin Like |
| *CAPZA1* | 0.30110275 | Capping Actin Protein Of Muscle Z-Line Subunit Alpha 1 |
| *CD300C* | -0.12117494 | CD300c Molecule |
| *CLIC1* | 0.30694692 | Chloride Intracellular Channel 1 |
| *FAM78A* | -0.02411514 | Family With Sequence Similarity 78 Member A |
| *GMFG* | -0.14743813 | Glia Maturation Factor Gamma |
| *GNG5* | 0.01316375 | G Protein Subunit Gamma 5 |
| *HPS3* | 0.07511752 | HPS3 Biogenesis Of Lysosomal Organelles Complex 2 Subunit 1 |
| *LRRC25* | -0.03352685 | Leucine Rich Repeat Containing 25 |
| *MFNG* | 0.15723002 | MFNG O-Fucosylpeptide 3-Beta-N-Acetylglucosaminyltransferase |
| *PLAUR* | 0.10091839 | Plasminogen Activator, Urokinase Receptor |
| *PLEKHA4* | 0.08055816 | Pleckstrin Homology Domain Containing A4 |
| *PLSCR1* | 0.13045411 | Phospholipid Scramblase 1 |
| *PTGER4* | -0.14440167 | Prostaglandin E Receptor 4 |
| *RAB38* | 0.08863284 | RAB38, Member RAS Oncogene Family |
| *RELB* | -0.16278248 | RELB Proto-Oncogene, NF-KB Subunit |
| *S100A10* | -0.04123815 | S100 Calcium Binding Protein A10 |
| *SPN* | 0.09415006 | Sialophorin |
| *TMSB4XP8* | 0.12615647 | Thymosin Beta 4 X-Linked Pseudogene 8 |
| *TNFRSF12A* | 0.20869316 | TNF Receptor Superfamily Member 12A |
| *TREM1* | -0.19778237 | Triggering Receptor Expressed on Myeloid Cells 1 |
| *TYROBP* | -0.09656099 | Transmembrane Immune Signaling Adaptor TYROBP |
